# Identification of SLC7A11‐AS1/SLC7A11 pair as a ferroptosis‐related therapeutic target for hepatocellular carcinoma

**DOI:** 10.1111/jcmm.18496

**Published:** 2024-07-10

**Authors:** Xiao Yuan, Yida Wang, Sitong Jiao, Huanhuan Gao, Mengqian Zhang, Xin Wang, Xunyu Zhou, Chuanfang Wu, Jinku Bao

**Affiliations:** ^1^ Key Laboratory of Bio‐Resource and Eco‐Environment of Ministry of Education, School of Life Science Sichuan University Chengdu China

**Keywords:** ferroptosis, hepatocellular carcinoma, lncRNA‐mRNA correlation network, SLC7A11, SLC7A11‐AS1

## Abstract

Hepatocellular carcinoma (HCC), a prevalent malignancy worldwide, poses significant challenges in terms of prognosis, necessitating innovative therapeutic approaches. Ferroptosis offers notable advantages over apoptosis, holding promise as a novel therapeutic approach for HCC complexities. Moreover, while the interaction between long non‐coding RNAs (lncRNAs) and mRNAs is pivotal in various physiological and pathological processes, their involvement in ferroptosis remains relatively unexplored. In this study, we constructed a ferroptosis‐related lncRNA‐mRNA correlation network in HCC using Pearson correlation analysis. Notably, the SLC7A11‐AS1/SLC7A11 pair, exhibiting high correlation, was identified. Bioinformatics analysis revealed a significant correlation between the expression levels of this pair and key clinical characteristics of HCC patients, including gender, pathology, Ishak scores and tumour size. And poor prognosis was associated with high expression of this pair. Functional experiments demonstrated that SLC7A11‐AS1, by binding to the 3′UTR region of SLC7A11 mRNA, enhanced its stability, thereby promoting HCC cell growth and resistance to erastin‐ induced ferroptosis. Additionally, in vivo studies confirmed that SLC7A11‐AS1 knockdown potentiated the inhibitory effects of erastin on tumour growth. Overall, our findings suggest that targeting the SLC7A11‐AS1/SLC7A11 pair holds promise as a potential therapeutic strategy for HCC patients.

## 1 INTRODUCTION

Hepatocellular carcinoma (HCC) stands among the most prevalent cancers worldwide and ranks as the third leading cause of cancer‐related deaths.^1^, ^2^ This prevalence is primarily attributable to challenges in early detection and resistance to standard chemotherapy treatments.^1^ Inducing apoptotic cell death with anticancer agents constitutes a primary strategy for eliminating cancer cells.^3^ However, the therapeutic efficacy of apoptosis‐based treatments is hampered by the evasion capabilities or resistance of cancer cells to apoptotic pathways,^4^ leading to the development of resistance following conventional chemotherapy.^3^ Sorafenib is the foremost choice among targeted therapeutic agents for clinical intervention in advanced liver cancer. Notably, recent research suggests that ferroptosis constitutes a primary mechanism underlying the anticancer effects of sorafenib.^5^, ^6^, ^7^ Therefore, a thorough investigation of the ferroptosis pathway is crucial.

Ferroptosis is an emerging form of programmed cell death resulting from the accumulation of iron‐dependent intracellular lipid peroxidation and is distinct from other PCDs.^8^ Based on the distinctive attributes of metabolic pathways, the chemical underpinnings of ferroptosis can be broadly categorized into three classes: iron metabolism, glutathione (GSH) metabolism and lipid metabolism.^9^ Several studies have also revealed that erastin (a ferroptosis inducer) exhibits comparable functionality to sorafenib in HCC, disrupting cystine uptake, inducing ferroptosis and promoting cell death in liver cancer by inhibiting the function of System Xc‐.^6^, ^10^ Importantly, SLC7A11, the primary executor of cystine/glutamate antiporter system Xc‐, plays a critical regulatory role in GSH metabolism.^11^, ^12^, ^13^ Moreover, SLC7A11 is often overexpressed in cancer, and its upregulation is necessary to adapt to high oxidative stress microenvironments and maintain cellular redox balance.^14^, ^15^ Therefore, targeting SLC7A11 to deplete cysteine may be an effective novel strategy for cancer treatment. Given the higher levels of reactive oxygen species (ROS) in cancer cells and their dependency on SLC7A11, targeting SLC7A11 to deplete cyst(e)ine could render cancer cells more susceptible to further oxidative stress.

Long non‐coding RNAs (LncRNAs) are a class of RNA molecules with a length exceeding 200 nucleotides that do not encode proteins.^16^ Notably, they play essential roles in epigenetic regulation, mRNA translation and protein modification,^22^ through interactions with DNA, RNA, or proteins.^17^ Notably, aberrant expression of LncRNAs is closely intertwined with the process of ferroptosis. P53RRA, SNAI3‐AS, NEAT1, GABPB1‐AS1 can induce ferroptosis,^18^, ^19^, ^20^, ^21^ while LINC0033, PVT, OIP5‐AS1 and other lncRNAs can inhibit ferroptosis.^22^, ^23^, ^24^ Therefore, targeting lncRNAs to promote ferroptosis and suppress HCC progression has become an expanding research focus.

To further enrich the functional understanding of lncRNAs in HCC, we established a ferroptosis‐related lncRNA/mRNA correlation network in HCC, focusing specifically on the SLC7A11‐AS1/SLC7A11 pair. Bioinformatic analysis revealed a robust correlation between the high expression of the pair and a poor overall survival. SLC7A11‐AS1 enhances the stability of SLC7A11 mRNA by forming an RNA–RNA double‐stranded structure, promoting SLC7A11 expression and thereby inhibiting ferroptosis and promoting tumorigenesis of HCC in vitro and vivo. In summary, by shedding light on the potential therapeutic role of ferroptosis, mainly through the lens of the SLC7A11‐AS1/SLC7A11 pair, our investigation provides a theoretical basis for the development of novel therapeutic targets for HCC.

## 2 MATERIALS AND METHODS

### 2.1 Data set

HCC gene expression data were derived from HCCDB (http://xsh.mywsat.cn/#/home).^25^ The transcriptome data from HCCDB25 and HCCDB30 were utilized for subsequent analyses. LncRNAs were annotated using the GENCODE database (https://www.gencodegenes.org). The TCGA database (https://portal.gdc. cancer.gov/repository) was used to get external HCC datasets. Finally, ferroptosis‐related genes were obtained from FerrDb V2 (http://www.zhounan.org/ferrdb/current/operations/help.html).^26^

### 2.2 Construction of ferroptosis‐related lncRNA/mRNA network

Using the ‘limma’ package in R, we set the parameters to identify ferroptosis‐related lncRNAs with an absolute correlation (|Cor|) exceeding 0.3 and a *p*‐value below 0.01. Additionally, the co‐expression relationships were visualized using Sankey diagrams and heatmaps. The Cytoscape software was also utilized to construct a correlation network based on these lncRNA‐mRNA pairs.

### 2.3 Clinical relevance analysis

The average expression value of these two genes was calculated as the SAS value for each sample. Subsequently, a series of correlation analyses were performed between SAS values and various clinical indicators, encompassing sex, tumour size, tumour analysis, immune infiltration, and survival prognosis. We used a heatmap to display the clinical feature correlations in the SLC7A11‐AS1/SLC7A11 pair. The chi‐square test analysed key clinical feature associations depicted in bar plots. Kaplan–Meier analysis assessed overall survival rates among HCC patients using the log‐rank test. The mean SAS value was used to differentiate between the high‐expression and low‐expression groups.

### 2.4 RNA–Seq and functional enrichment analysis

RNA extraction for RNA‐Seq was conducted on HepG2 hepatoblastoma cells from either the knockdown group or the control group. The high‐throughput RNA sequencing was conducted by Novogene (Beijing, China). Raw data from Illumina HiSeq sequencing underwent filtration and alignment against a reference sequence using TopHat2. Thus, forming the basis for quantification analysis of known and novel genes. We also utilized DESeq to pinpoint differentially expressed genes (DEGs).^27^ The sequencing data related to this study has been stored in the National Center for Biotechnology Information's Gene Expression Omnibus (GEO) under the accession number GSE244762. Furthermore, analyses of the G.O. and KEGG pathways were conducted following methods previously described.^28^, ^29^

### 2.5 Cell cultures

Human HCC cell lines, namely HepG2, Huh7, Hep3B, 97H, and LM3, were procured from the Institute of Biochemistry and Cell Biology, Chinese Academy of Sciences (Shanghai, China) in 2019. All cells were cultivated in Dulbecco's Modified Eagle Medium (Gibico, USA) with 10% FBS and maintained at 37°C in a 5% CO_2_ environment. Authentication via short tandem repeat (STR) profiling was performed for all cell lines, and monthly assessment using a mycoplasma detection kit (Beyotime, Shanghai, China) was conducted to ensure the absence of mycoplasma contamination in the cell cultures.

### 2.6 Vectors construction and lentiviral infection

The full‐length SLC7A11‐AS1 transcriptional sequence was constructed by Genewiz (Suzhou, China) and cloned into the pLVX‐IRES lentiviral vector. Overlapping and non‐overlapping regions of the SLC7A11‐AS1 transcriptional sequence were synthesized based on pLVX‐SLC7A11‐AS1 using Phanta Super‐Fidelity DNA Polymerase (Vazyme, Jiangsu, China). The full‐length SLC7A11 transcription sequence was synthesized from HepG2 cDNA and cloned into the expression vector pLVX lentiviral vector. The pLKO.1 lentiviral vector was also employed to construct shRNA. Primer sequences are presented in Table S1. The target plasmid and packaging plasmids were co‐transfected into 293 T cells using Lipofectamine 3000 (Thermo Fisher, USA) for virus packaging. Following 48 h of transfection, the supernatant was collected, filtered, and employed for the infection experiments.

### 2.7 Real‐time polymerase chain reaction (qRT‐PCR)

Cell lysis was carried out using a TRIzol reagent, followed by total RNA extraction. Reverse transcription was performed using a Reverse Transcriptase (Yeasen, Shanghai, China) and detected using qPCR SYBR Green Master Mix (Yeasen) according to the product specification. The primer sequences utilized for qRT‐PCR are provided in Table S1.

### 2.8 Cell‐proliferation assay

#### 2.8.1 Cell counting kit‐8 assay

Cells were plated at 8000 cells per well in a 96‐well plate, with each sample group simultaneously seeded on five separate plates. Following the instructions, the Counting Kit‐8 (CCK8) (Beyotime) was employed, with daily measurements conducted over a consecutive period of 5 days.

#### 2.8.2 Colony‐formation assay

Cells were seeded at a density of 100 cells per well in a 6‐well plate. After 14 days, single‐cell colonies were fixed, stained, and washed. Subsequently, images were captured, and the number of colonies was counted.

### 2.9 Cell cycle analysis

Following cellular treatment, fixation was performed at 4°C for 12 h using 70% ethanol. Subsequently, cells were stained using the Cell Cycle and Apoptosis Analysis Kit (Beyotime). At least 10,000 cells were acquired on LSRfortessa (B.D., USA), and FlowJo analysis was employed to compute the percentages of cells in the G1, S or G2/M phases of the cell cycle.

### 2.10 Measurement of L‐ROS, GSH level and MDA level

#### 2.10.1 L‐ROS measurement

L‐ROS levels were quantified using BODIPY‐C11 (Thermo Fisher) dye and analysed by flow cytometry. Cells were plated at a density of 2.5×10^5^ cells per well in a 6‐well plate and subjected to appropriate treatments. Following treatments, cells were incubated with 5 μM BODIPY‐C11 in the dark at 37°C for 45 min. Subsequently, cells were triple‐washed with PBS, resuspended in 500 μL PBS, and subjected to flow cytometry for fluorescence intensity measurement.

#### 2.10.2 GSH assay

The relative GSH concentration in samples was assessed with the Glutathione Assay Kit (Solabio, Beijing, China). Cells were seeded at 5 × 10^6^ cells per 10 cm culture dish and processed per the GSH assay kit's instructions.

#### 2.10.3 MDA assay

Cellular lysates were gauged via the Lipid Peroxidation Assay Kit (Solabio). Cells were seeded at 5×10^6^ cells per 10 cm culture dish. Cells were processed per the MDA assay kit's protocol.

### 2.11 Western blot

Cells were lysed using RIPA lysis buffer (Beyotime) supplemented with 1% PMSF. Subsequently, protein samples were separated using SDS‐PAGE gel and transferred onto a PVDF membrane (Millipore, USA). After blocking, the membrane was incubated with the primary antibody overnight at 4°C. Then, the membrane was incubated with the second antibody for 1 h after washing. Following incubation, the membrane was treated with an enhanced chemiluminescent (ECL) reagent (Vazyme) and visualized using a ChemiDoc Touch system (Bio‐Rad, USA). The antibody details are available in Table S2.

### 2.12 Fluorescence in suit hybridization (FISH)

Probes for SLC7A11‐AS1 and SLC7A11 were synthesized by Gene Pharma (Jiangsu, China) and are presented in Table S1. Following fixation and subsequent washing, FISH analysis was conducted using specific probes and a FISH kit (Gene Pharma, Jiangsu, China). Image capture was performed using the SpinSR10 confocal microscope (Olympus, Japan).

### 2.13 RNA–RNA pulldown assay

Transcription of SLC7A11‐AS1 sense/antisense strands from pLVX‐SLC7A11‐AS1 was performed in vitro using T7 RNA polymerase (Yeasen). Following digestion of DNA and purification, the resulting RNA was biotinylated using the Pierce™ RNA 3′ End Biotinylation Kit (Thermo Fisher). Subsequently, 5 μg of biotinylated probes were incubated with 1 mg of HepG2 cell lysate at 4°C for 2 h. Following this, 100 μL of Dynabeads™ M‐270 (Thermo Fisher) were added and incubated overnight. Finally, the enriched RNA was subjected to RT‐qPCR analysis.

### 2.14 Xenograft assay and immunohistochemistry (IHC)

Female BALB/C nude mice aged 4–6 weeks were purchased from GemPharmatech (Jiangsu, China) and were kept under a sterile specific pathogen‐free (SPF) environment. Each mouse was subcutaneously injected with 5 × 10^6^/100 μL HepG2 cells. One week following injection, tumour volume was measured every 3 days. Regarding erastin treatment, 21 days after injection, mice were intraperitoneally injected with 5 mg/mL erastin or DMSO every 3 days. The Animal Care and Use Committee of Sichuan University approved all experimental protocols.

Tumour samples were fixed overnight in formalin solution, dehydrated in ethanol, embedded in paraffin, and subsequently sectioned. These sections were further subjected to staining with haematoxylin and eosin (H&E) for histological assessment and immunohistochemical analysis for SLC7A11.

### 2.15 Statistical analysis

Statistical analyses were executed using the SPSS software package. Graphical visualization was undertaken using GraphPad Prism software. In the absence of specific indications, it is noteworthy that a minimum of three biological replicates were performed for each experiment, thus encompassing biological diversity. Differences in statistical measures between groups were assessed using the *t*‐test and analysis of variance (ANOVA) methodologies. In the context of significance levels, a *p*‐value less than 0.05 is denoted by *, less than 0.01 by **, and less than 0.001 by ***.

## 3 RESULTS

### 3.1 Construction of ferroptosis‐related lncRNA‐mRNA networks

**FIGURE 1:**
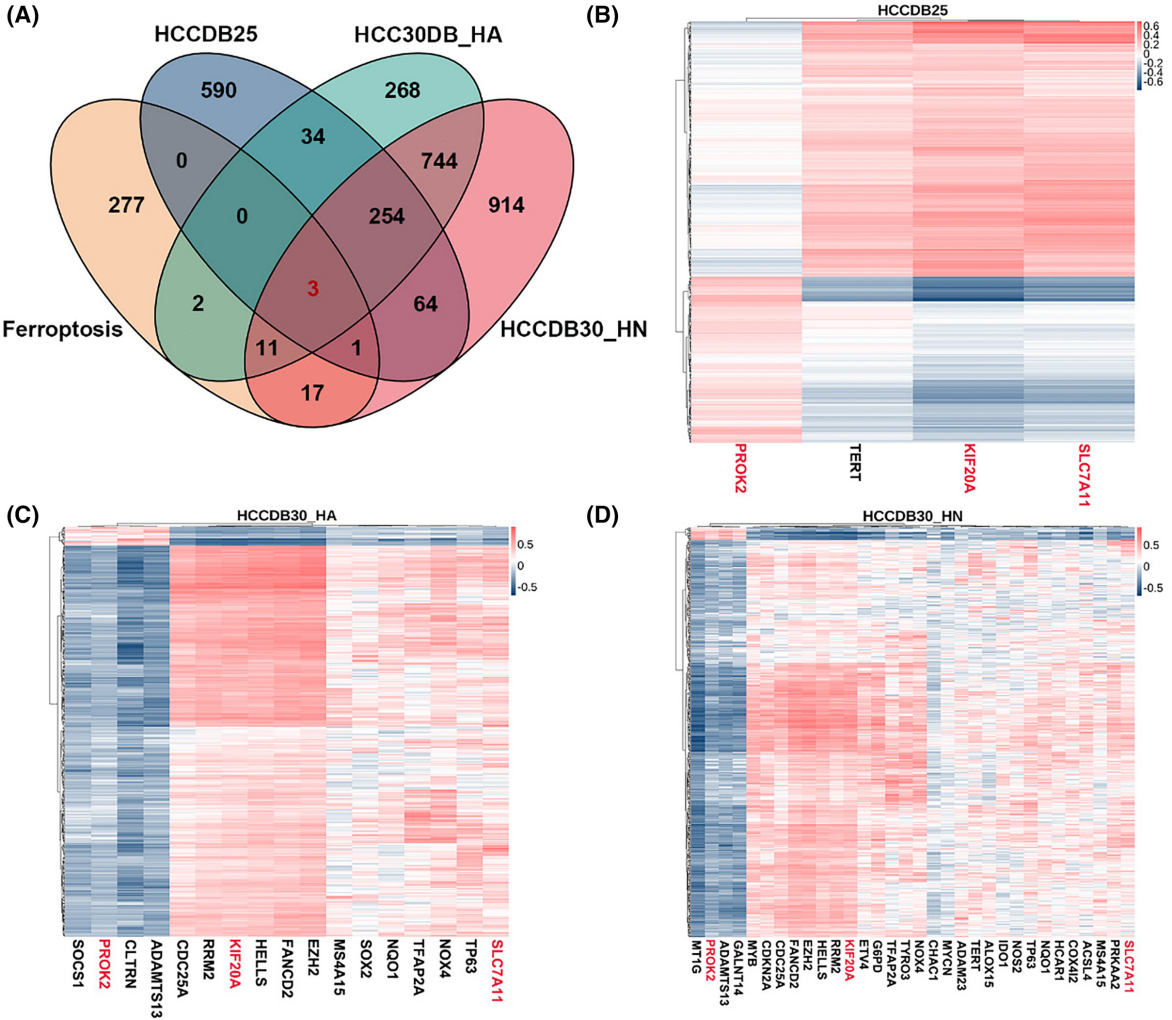
Identification of ferroptosis‐related differentially expressed mRNAs and lncRNAs in HCC patients. (A) The Venn diagram illustrates the common DEmRNA between three datasets and ferroptosis‐related genes. (B–D)The heatmap depicts the correlation between lncRNAs and ferroptosis‐related mRNAs in HCCDB25 (B), HCCDB30_HA (C), and HCCDB30_HN (D).

To validate differentially expressed lncRNAs and mRNAs associated with ferroptosis in HCC, we acquired two datasets from HCCDB (HCCDB25 and HCCDB30). Due to the inclusion of diverse sample types in HCCDB30, we divided them into HCCDB30_HA (cancer and adjacent‐cancer data) and HCCDB30_HN (cancer and normal data) to facilitate a more precise analysis. The volcano plot illustrates differentially expressed lncRNAs (DElncRNAs) and differentially expressed mRNAs (DEmRNAs) across these three datasets (Figure S1A–C). Firstly, we selected ferroptosis‐related DEmRNAs associated with ferroptosis with high confidence levels from FerrDb. The Venn diagram also demonstrates the intersection of DEmRNAs with ferroptosis‐related genes across these three datasets (Figure 1A). A total of 35 ferroptosis‐related mRNAs were identified, with KIF20A, SLC7A11, and PROK2 emerging as common ferroptosis‐related mRNAs across all three datasets. Based on these ferroptosis‐related mRNAs, we identified 695 differentially expressed lncRNAs (DElncRNAs) through Pearson correlation analysis, serving as ferroptosis‐related lncRNAs(Figure 1B–D).

**FIGURE 2:**
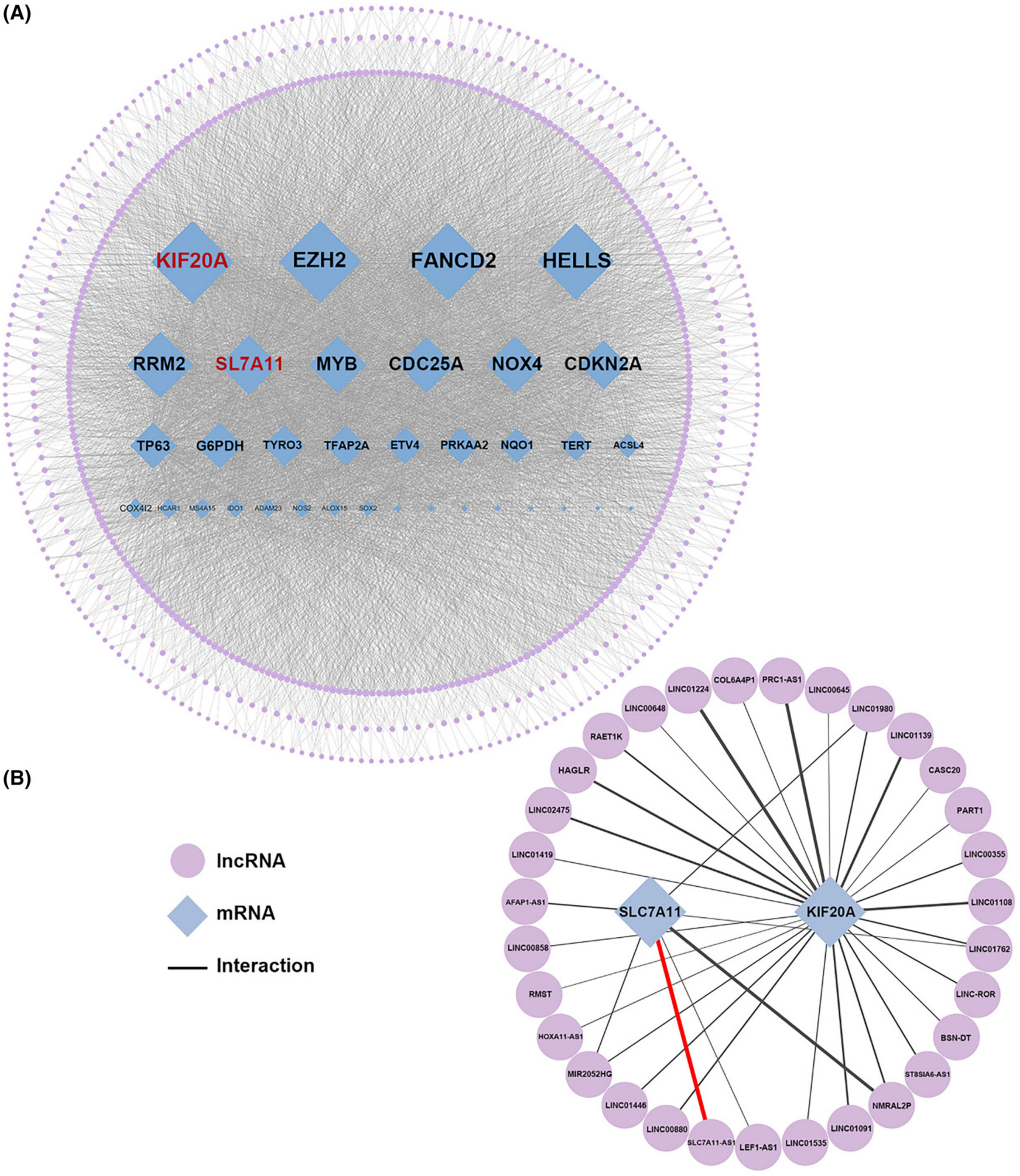
Construction of ferroptosis‐related lncRNA‐mRNA correlation network based on person correlation. (A) Constructed lncRNA‐mRNA Correlation Network based on all ferroptosis‐related mRNA and lncRNA from three datasets. The lncRNAs were also organized into three concentric layers based on their node degree, ranging from 1 to 5, 6–10 and 11–35 from the outermost to the innermost layer. (B) Constructed lncRNA‐mRNA Correlation Network based on common ferroptosis‐related mRNA and lncRNA across three datasets.

We then constructed a ferroptosis‐related lncRNA‐mRNA correlation network (Figure 2A). The network comprises 730 nodes, including 35 mRNAs and 695 lncRNAs, with a total of 6344 edges. The shared portion among the three datasets is illustrated in greater detail in Figure 2B, where the thickness of the edges positively correlates with the strength of the correlation (Table S3). In this shared network, SLC7A11 and KIF20A emerge as central mRNAs, underscoring their pivotal roles in ferroptosis in HCC. Notably the SLC7A11‐AS1/SLC7A11 pair exhibited a high correlation (HCCDB25: *r* = 0.380; HCCDB30_HA: *r* = 0.914; HCCDB30_HN: *r* = 0.914), indicating a strong association between these genes in the context of HCC. Therefore, we chose the correlation network based on the SLC7A11‐AS1/SLC7A11 pair as the focus for subsequent investigations.

### 3.2 Clinical correlation analysis of the SLC7A11‐AS1/SLC7A11 pair in HCC

**FIGURE 3:**
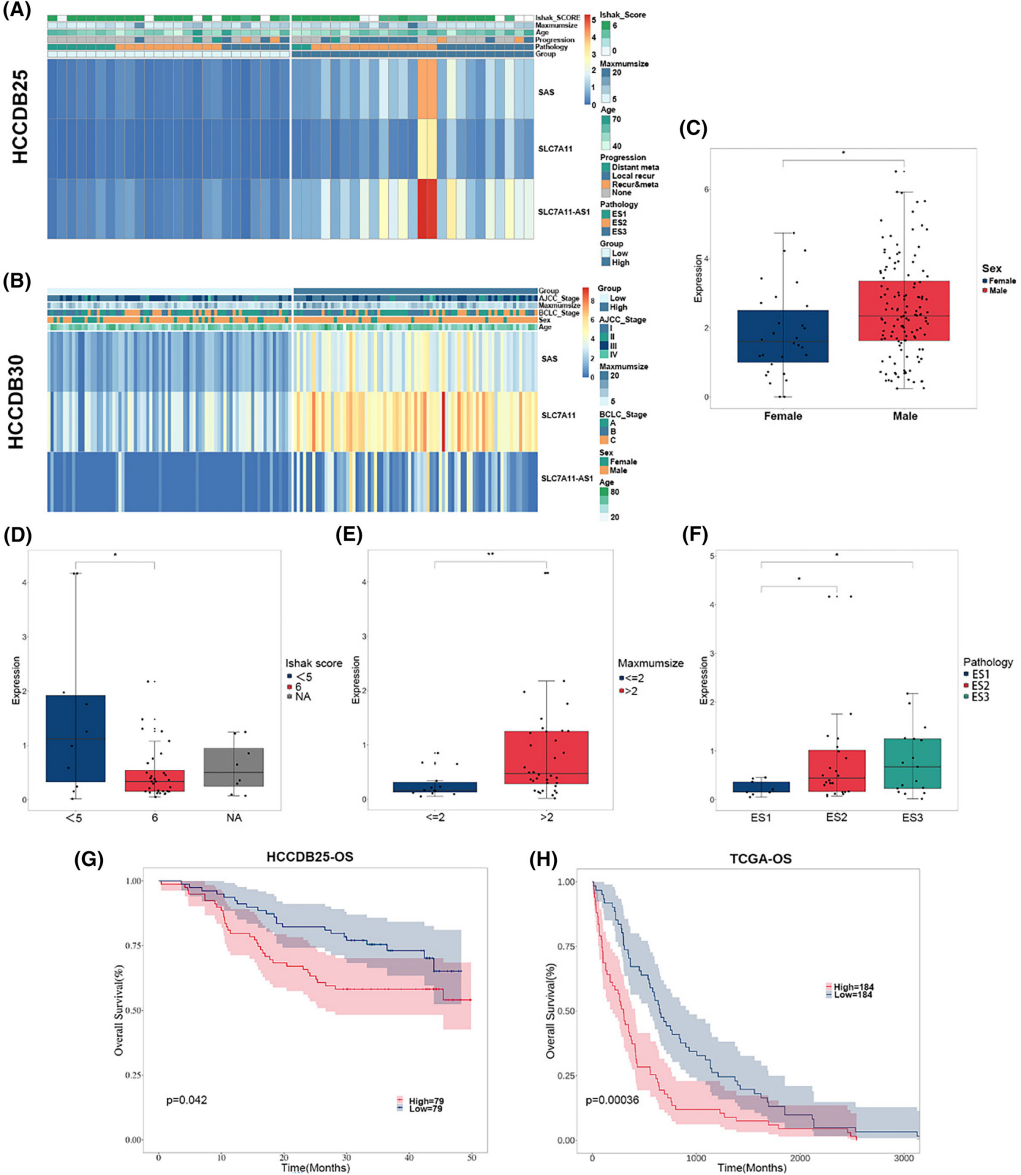
Clinical relevance of SLC7A11‐AS1/SLC7A11 correlation network. (A, B**)** Heatmap of correlation for the risk model in HCCDB25 (A) and HCCDB30 (B). (C–F**)** The boxplot demonstrates the relationship between certain clinical features and the expression levels of the SLC7A11‐AS1/SLC7A11 pair in HCCDB25, including gender (C), pathology (D), Ishak score (E), and maximum size (F). (G, H) Kaplan–Meier curve analysis was conducted to examine the overall survival rates of patients with high and low expression of the SLC7A11‐AS1/SLC7A11 pair in HCCDB25 (G) or TCGA‐LIHC (H). SAS, SLC7A11‐AS1/SLC7A11 correlation network.

To assess the clinical significance of the identified correlation network based on the SLC7A11‐AS1/SLC7A11 pair in HCC, we categorized the model into high and low‐expression groups. These were categorized at a 1:1 ratio based on the expression levels of SLC7A11‐AS1/SLC7A11, conducting comprehensive analyses of clinical‐pathological features and survival rates for both groups. Notably, the expression levels of this pair showed a significant association with various clinical‐pathological characteristics in HCC patients, including gender (HCCDB25), pathology, Ishak score and maximum size (HCCDB30) (Figure 3A–F). These findings also indicate the SLC7A11‐AS1/SLC7A11 pair's capacity to effectively differentiate patients within distinct clinical subgroups. Moreover, Kaplan–Meier survival analysis in HCCDB25 indicated a marked reduction in overall survival rates for patients with high SLC7A11‐AS1/SLC7A11 expression compared to those with low expression (Figure 3G). Likewise, Kaplan–Meier curve analysis in TCGA‐LIHC also confirms that high expression of SLC7A11‐AS1/SLC7A11 is associated with shorter overall survival (Figure 3H). Thus, this suggests that the SLC7A11‐AS1/SLC7A11 pair could serve as a molecular marker for HCC.

### 3.3 Functional enrichment analysis of the SLC‐7A11‐AS1/SLC7A11 pair in HCC

**FIGURE 4:**
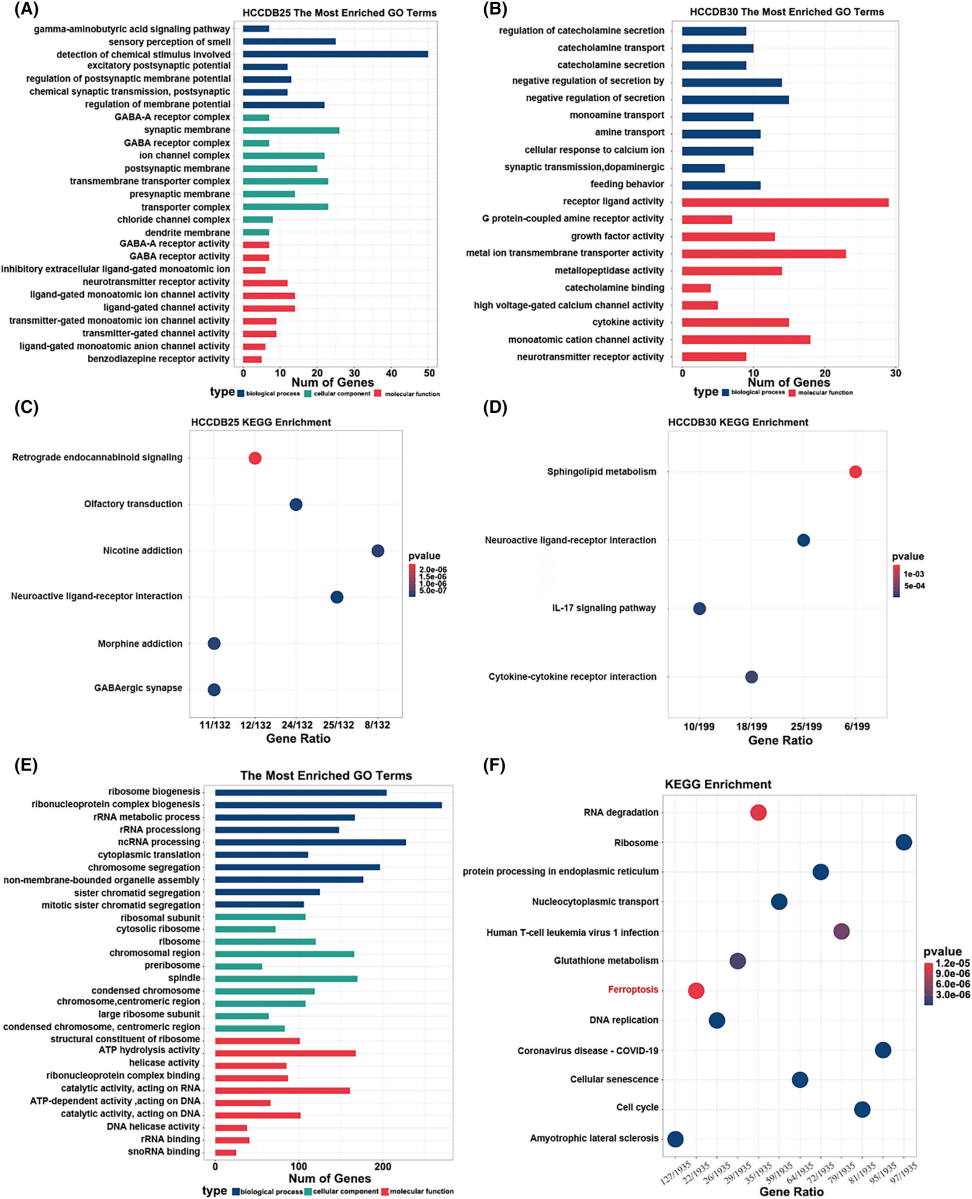
Functional enrichment analysis of the SLC7A11‐AS1/SLC7A11 correlation network. (A, B) Bar graph showing enriched G.O. terms of HCCDB25 (A) and HCCDB30 (B). (C, D) Bar plot showing the enriched KEGG pathways of HCCDB25(C) and HCCDB30 (D). (E, F) Enriched G.O. terms (E) and enriched KEGG pathways (F) from RNA‐Seq results of SLC7A11‐AS1 knockdown.

We performed a functional enrichment analysis to uncover the molecular mechanisms distinguishing the high and low expression groups of the SLC7A11‐AS1/SLC7A11 pair in HCC. The enriched G.O. terms identified in the HCCDB25 and HCCDB30 datasets include the detection of chemical stimulus, ion channel complex, metal ion transmembrane transporter activity, monoatomic cation channel activity, cytokine activity, and negative regulation of secretion (Figure 4A,B). Additionally, KEGG enrichment analysis identified involvement in pathways such as neuroactive ligand‐receptor interaction, GABAergic synapse, sphingolipid metabolism, IL‐17 signalling and cytokine‐cytokine receptor interaction, suggesting these pathways' potential roles in the pathogenesis of HCC or its response to treatment (Figure 4C,D).

Current research on SLC7A11 is extensive, with numerous studies confirming its role in regulating diverse physiological activities, including redox status, ferroptosis, and intercellular signalling.^11^, ^30^ An analysis of SLC7A11‐AS1's expression levels was conducted using HCC cells, explicitly encompassing Hep3B, LM3, Huh‐7, HepG2 and 97H cell lines (Figure S2A). Then, we stably knocked down SLC7A11‐AS1 in three cell lines (HepG2, HuH‐7 and LM3) with relatively high SLC7A11‐AS1 expression (Figure S2B). We then performed RNA sequencing in HepG2 cells with SLC7A11‐AS1 knockdown, revealing 2536 DEGs between the sh‐Control group and shSLC7A11‐AS1#1 group (Figure S2C). G.O. enrichment analysis revealed that the DEGs are predominantly involved in ribonucleoprotein complex biogenesis, ncRNA processing, chromosomal region, and catalytic activity (Figure 4E). Furthermore, KEGG enrichment analysis further indicated a close association of these DEGs with signalling pathways relevant to cancer development (Figure 4F). Additional details are presented in Figure S2D). Therefore, these results suggest that SLC7A11‐AS1 may influence tumour ferroptosis and impact the progression of HCC.

### 3.4 SLC7A11‐AS1 promoting HCC cell growth and suppressing ferroptosis through positively regulating SLC7A11

**FIGURE 5:**
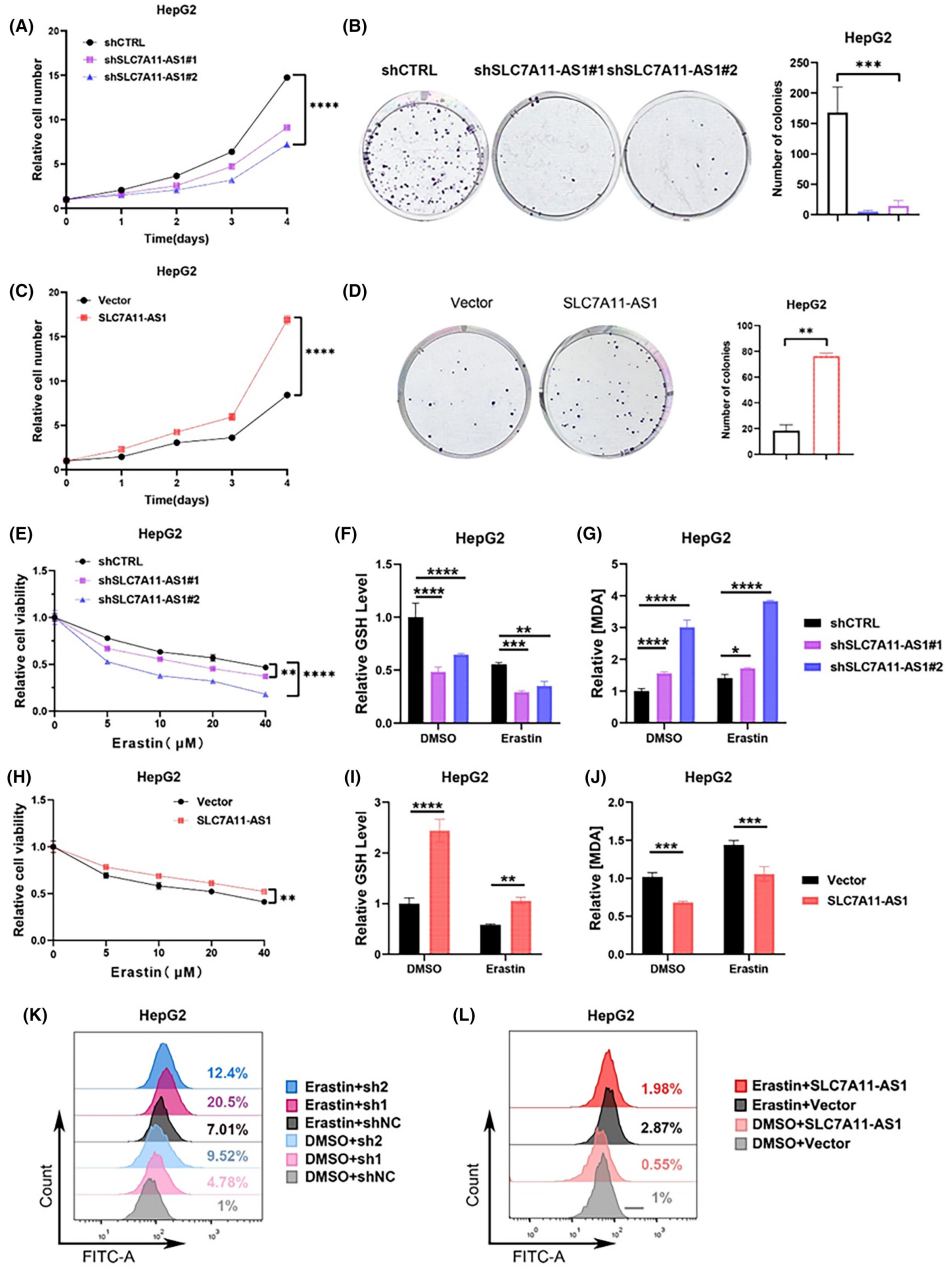
SLC7A11‐AS1 promotes cell proliferation and suppresses ferroptosis in HepG2. (A, B) After knocking down SLC7A11‐AS1 in HepG2 cells, cell proliferation was assessed using the CCK8 assay (A), and clonogenic formation ability was evaluated (B). (C, D) After overexpression SLC7A11‐AS1 in HepG2 cells, cell proliferation was assessed using the CCK8 assay (C), and clonogenic formation ability was evaluated (D). (E) CCK8 assay to determine cell viability after treatment with gradient concentrations of erastin for 24 h in SLC7A11‐AS1 knockdown cells. (F, G) HepG2 cells knocking‐down SLC7A11‐AS1 were treated with erastin for 24 h, GSH levels were measured using a glutathione assay kit (F), lipid peroxidation was assessed by measuring MDA levels (G). (H) CCK8 assay to determine cell viability after treatment with gradient concentrations of erastin for 24 h in SLC7A11‐AS1 overexpressing cells. (I, J) HepG2 cells overexpression SLC7A11‐AS1 were treated with erastin for 24 h, GSH levels were measured using a glutathione assay kit (I), lipid peroxidation was assessed by measuring MDA levels(J). (K, L) the levels of L‐ROS in HepG2 cells knocking‐down (K) or overexpression (L) SLC7A11‐AS1 were detected using flow cytometry with C11‐BODIPY staining. Data are presented as mean ± S.D. of three independent experiments. *p < 0.05, **p < 0.01, ***p<0.001, ****p<0.0001, ns: not significant.

The tumorigenic phenotypes of HepG2, HuH‐7 and LM3 cells were examined using a stable knockdown of SLC7A11‐AS1 to investigate the function of SLC7A11‐AS1 in liver cancer. CCK‐8 assays demonstrated that the shSLC7A11‐AS1 group exhibited lower cell viability than the shControl group (Figure 5A). Likewise, the clonogenic ability of the shSLC7A11‐AS1 group was significantly suppressed (Figure 5B). Moreover, overexpression of SLC7A11‐AS1 was found to enhance the viability and clonogenic ability of HepG2 cells (Figure 5C,D), as the same results were also obtained in HuH‐7 and LM3 cell lines (Figure S3). Thus, these experiments suggest that SLC7A11‐AS1 can promote the proliferation of liver cancer cells.

Subsequently, we investigated whether SLC7A11‐AS1 regulates ferroptosis in liver cancer cells. CCK8 assay indicated that the shSLC7A11‐AS1 group was more sensitive to erastin‐induced ferroptosis than the shContrl group (Figure 5E). Importantly, the elevation of L‐ROS and MDA, along with the decrease in GSH, are crucial indicators of ferroptosis occurrence [32]. Thus, we measured these three indicators in liver cancer cells with downregulated SLC7A11‐AS1. As expected, the results showed that the GSH content in the shSLC7A11‐AS1 group was significantly reduced (Figure 5F), and the MDA content was significantly increased (Figure 5G). Moreover, flow cytometry results demonstrated that the depletion of SLC7A11‐AS1 in HCC cells significantly increased the intracellular L‐ROS content. Furthermore, using erastin treatment in shContrl or shSLC7A11‐AS1 cells for 24 h increased the accumulation of L‐ROS induced by erastin in the absence of SLC7A11‐AS1(Figure 5K). On the contrary, overexpression of SLC7A11‐AS1 diminished the sensitivity of HepG2 cells to erastin (Figure 5H), elevated intracellular levels of GSH (Figure 5I), and concurrently significantly decreased the levels of intracellular MDA (Figure 5J). The flow cytometry results also showed that the upregulation of SLC7A11‐AS1 expression inhibited the accumulation of intracellular L‐ROS induced by erastin (Figure 5L). Similarly, we assessed the impact of SLC7A11‐AS1 on ferroptosis in Huh7 and LM3 cells, obtaining results consistent with those observed in HepG2 cells (Figure S4).

We then investigated cell cycle variations according to the indications from the KEGG enrichment analysis in Figure 3F. Flow cytometry outcomes revealed that the suppression of SLC7A11‐AS1 in HepG2 cells resulted in an augmentation of the cell population within the G1 phase, concomitant with a reduction in the proportion of cells occupying the S phase. As such, these suggest that SLC7A11‐AS1 depletion in HepG2 cells potentially hinders the transition of cells from the G1 phase to the S phase. However, the downregulation of SLC7A11‐AS1 did not notably influence the cell cycle dynamics in Huh7 and LM3 cells (Figure S5A,B). Furthermore, the overexpression of SLC7A11‐AS1 did not affect the cell cycle (Figure S5C,D).

**FIGURE 6:**
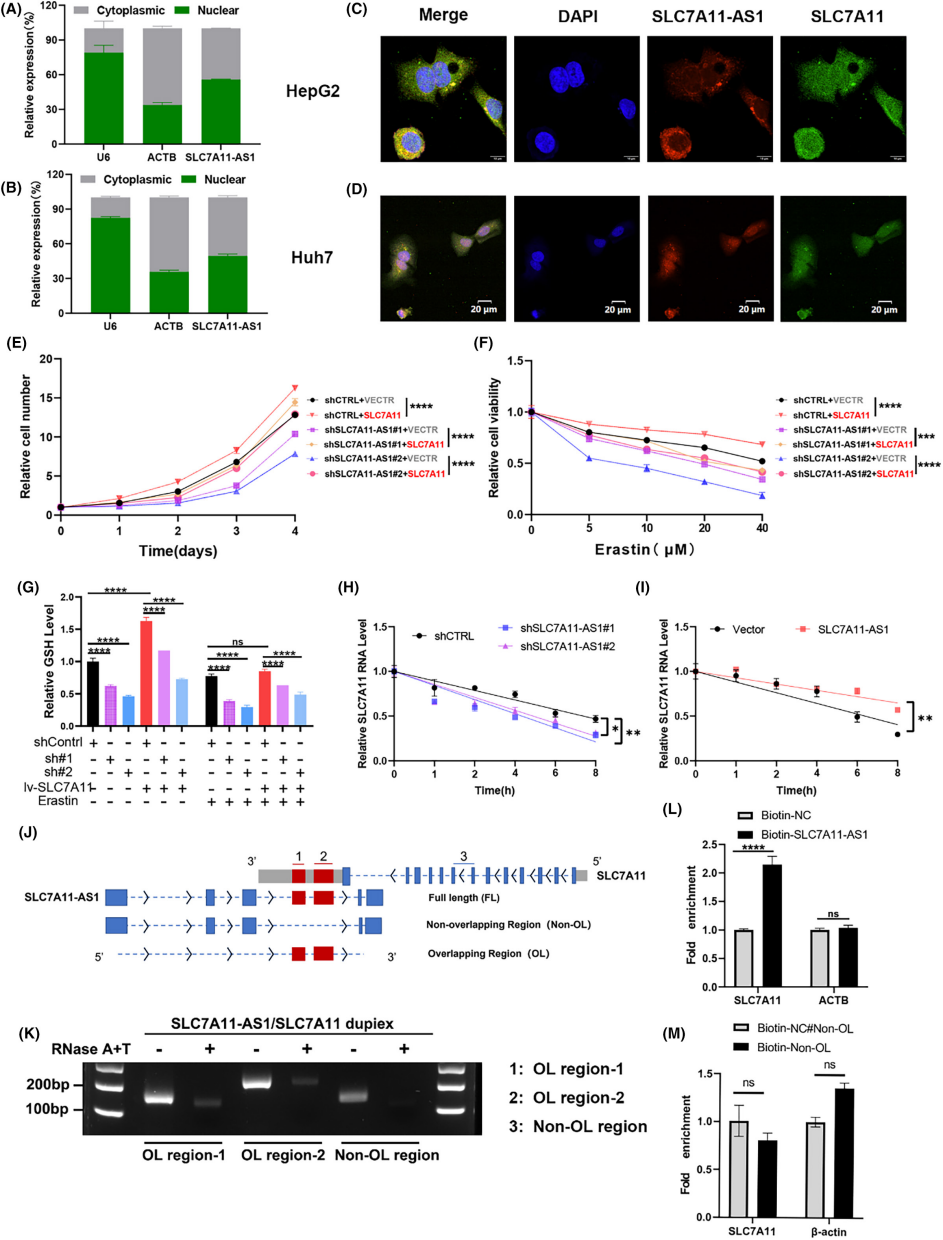
SLC7A11‐AS1 binds to SLC7A11 mRNA, enhancing its stability. (A, B) The SLC7A11‐AS1 expression levels in the nuclear and cytosolic fractions derived from HepG2 (A) and HuH7 (B) cells by qRT‐PCR analysis. (C, D) FISH analysis was performed to evaluate the colocalization of endogenous SLC7A11‐AS1 and SLC7A11 RNA in HepG2(C) and HuH7 cells (D). (E) Cell proliferation of each group was measured using CCK8 assay. (F) CCK8 assay was used to measure cell viability of each group after treatment with varying concentrations of erastin for 24 h. (G) Glutathione assay kit measured GSH levels in each group after 24 h of erastin treatment. (H, I) The stability of SLC7A11 mRNA over time was detected by qRT‐PCR relative to time 0 after blocking new RNA synthesis with Actinomycin D (20 μg/mL) in HepG2 cells after knockdown of SLC7A11‐AS1 (H) or overexpression of SLC7A11‐AS1 (I). RNA levels were normalized to 18S rRNA. (J) Structural diagram of SLC7A11‐AS1 and SLC7A11 RNA, as well as a schematic representation of the full length, overlapping region, and non‐overlapping region of SLC7A11‐AS1. The red region indicates the overlapping region between SLC7A11 and SLC7A11‐AS1. 1: Overlapping region 1, 2: Overlapping region 2, 3: non‐overlapping region. (K) RNase protection assay was performed on RNA samples from HepG2 cells, and PCR amplification was used to detect the O.L. region‐1, O.L. region‐2, and the non‐O.L. regions of SLC7A11. (L, M) The RNA–RNA interaction between the F.L. (L) and non‐O.L. region (M) of SLC7A11‐AS1 transcript and SLC7A11 mRNA was detected by RNA pulldown assay. Data are presented as mean ± S.D. of three independent experiments. *p < 0.05, **p < 0.01, ***p<0.001, ****p<0.0001, ns, not significant.

Experiments conducted on HCC cells confirmed the oncogenic effects of SLC7A11‐AS1 and its inhibitory role in ferroptosis. Subsequently, we focused on elucidating the underlying mechanisms of the SLC7A11‐AS1/SLC7A11‐associated network. Our results showed that SLC7A11‐AS1 is distributed in both the nucleus and cytoplasm, with approximately 44% of SLC7A11‐AS1 located in the cytoplasm in HepG2 cells and 50% in HuH‐7 cells (Figure 6A,B). FISH colocalization analysis also showed that SLC7A11‐AS1 and SLC7A11 co‐localized in the cytoplasm (Figure 6C,D). Subsequently, a rescue experiment was conducted to confirm whether SLC7A11‐AS1 functions through SLC7A11 (Figure S6A). Notably, CCK‐8 assays showed that the overexpression of SLC7A11 partially rescued the growth inhibition caused by the depletion of SLC7A11‐AS1 (Figure 6E) and partially restored the increased sensitivity to erastin induced by the knockdown of SLC7A11‐AS1 (Figure 6F). The decrease in GSH levels caused by knocking down SLC7A11‐AS1 can also be partially restored by overexpressing SLC7A11(Figure 6G). Thus, these results suggest that SLC7A11‐AS1 promotes liver cancer growth by positively regulating ferroptosis through SLC7A11.

### 3.5 SLC7A11‐AS1 promotes the stability of SLC7A11 mRNA via mutual binding

SLC7A11‐AS1 is in the ‘tail‐to‐tail’ fashion with the SLC7A11 antisense strand. Previous studies have shown that antisense RNAs can form RNA–RNA duplexes with their sense mRNAs. These complexes can avoid ribonucleases degradation, thereby altering the mRNA half‐life.^31^, ^32^ The impact of SLC7A11‐AS1 on the stability of SLC7A11 mRNA was subsequently evaluated by treating cells with α‐amanitin to inhibit new RNA synthesis within 8 h. RT‐qPCR results indicated a significant reduction in SLC7A11 mRNA stability upon SLC7A11‐AS1 knockdown (Figure 6H). At the same time, the overexpression of SLC7A11‐AS1 significantly increased the stability of SLC7A11 mRNA (Figure 6I). Consistent results were also obtained in Huh7 cells (Figure S6B,C). Furthermore, treating HCC cells with protein synthesis inhibitor CHX and proteasome inhibitor MG132 showed that alterations in SLC7A11‐AS1 expression did not affect the synthesis or degradation of the SLC7A11 protein (Figure S6D,E). These results indicate that SLC7A11‐AS1 primarily regulates SLC7A11 at the RNA level.

Sequence alignment results further demonstrated a high homology between exon 4 and exon 5 of SLC7A11‐AS1 and the 3’ UTR region of SLC7A11. The RNase protection assay (RPA) was employed to verify whether the formation of the RNA duplex depends on the overlapping region.^33^ To conduct this study, probes were formulated to specifically recognize regions on SLC7A11 that either overlap (O.L.) or do not overlap (non‐O.L.) with SLC7A11‐AS1 (Figure 6J). The outcomes of PCR amplification distinctly revealed that the non‐O.L. region underwent complete digestion by RNase. In contrast, the O.L. region exhibited partial protection against degradation (Figure 6K). The RNA–RNA pulldown results further confirmed that non‐OL could not specifically bind to SLC7A11 mRNA, unlike F.L. (full‐length) SLC7A11‐AS1 (Figure 6L,M). Notably, the binding between SLC7A11‐AS1 and SLC7A11 mRNA primarily relies on the overlapping region.

Then, we postulated that the significance of the overlapping region within SLC7A11‐AS1 surpasses that of other segments. The non‐O.L. vector did not induce an elevation in the levels of SLC7A11 RNA and protein levels (Figure S6F,G). In contrast, the F.L. vector of SLC7A11‐AS1 markedly augmented the levels of SLC7A11 RNA and protein. However, the overexpression of the O.L. vector did not increase the SLC7A11 RNA and protein levels in cells. Moreover, overexpression of the O.L. region led to a significant decrease in the RNA level of SLC7A11‐AS1 (Figure S6H,I). Thus, these findings imply that the overlapping area is a necessary condition for the function of SLC7A11‐AS1 but not a sufficient condition.

### 3.6 SLC7A11‐AS1 promotes tumour growth in vivo

**FIGURE 7:**
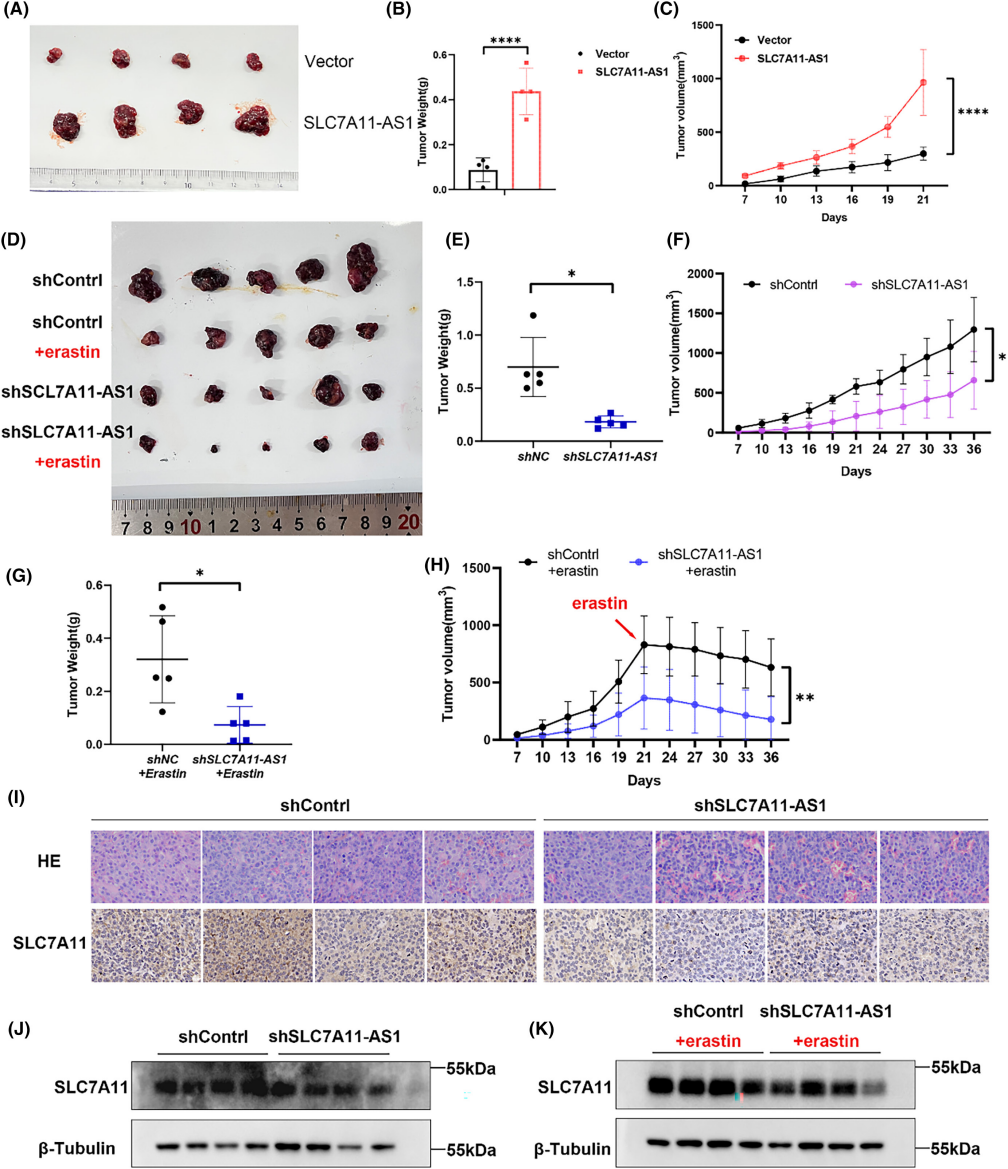
SLC7A11‐AS1 promotes tumour growth of cell xenografts in nude mice. (A–C) HepG2 cells from the SLC7A11‐AS1 group or Vector group were injected into nude mice(A), the tumour volumes were measured every 3 days after the cells were injected for 7 days(B), the tumour weight was recorded(C) (*n* = 5). (D) 5 HepG2 cells from the shSLC7A11‐AS1 group or shNC group were injected into nude mice and treated with erastin after 21 days or not. (E, F) Measure and count the weight of the tumour from nude mice not treated with erastin (E)or treated with erastin (F) (*n* = 5). (G, H) The tumour volumes were measured every 3 days after the cells were injected for 7 days (*n* = 5). (I) IHC detected SLC7A11 expression. (J, K) Western blot analysis of SLC7A11 protein levels in tumour tissues and tumour tissues after erastin treatment. Data are presented as mean ± S.D. of three independent experiments. *p < 0.05, **p < 0.01, ***p<0.001, ****p<0.0001, ns: not significant.

Overexpression of SLC7A11‐AS1 significantly enhanced the tumorigenicity of HepG2 cells in nude mice (Figure 7A–C). Additionally, significantly diminished tumour volume and weight were observed within the shSLC7A11‐AS1 group (Figure 7D–F). The subcutaneous tumour mouse model was treated with erastin to further validate the association between SLC7A11‐AS1 and ferroptosis in vivo. The results showed that erastin treatment yielded a therapeutic on both the shContrl and shSLC7A11‐AS1 groups. However, the therapeutic effect was more significant in the shSLC7A11‐AS1 group, consistent with our previous cell experiment results (Figure 7G,H), indicating that the attenuation of SLC7A11‐AS1 can enhance susceptibility to erastin‐triggered ferroptosis. Moreover, it was observed that the cell number was lower in the shSLC7A11‐AS1 group compared to the shContrl group. The expression level of SLC7A11 also exhibited a reduction (Figure 7I). Western blot analysis further confirmed a significant decrease in the expression level of SLC7A11 within the shSLC7A11‐AS1 group when contrasted with the shControl group (Figure 7J,K). In line with the cellular experiments, in vivo experiments also validated that downregulating SLC7A11‐AS1 enhances the sensitivity of HCC tumours to erastin.

## 4 DISCUSSION

Accumulating studies have revealed the potential of targeting ferroptosis for clinical treatment of HCC, making a deeper understanding of the molecular basis of ferroptosis crucial for HCC therapy.^34^, ^35^ Many previous studies have emphasized the importance of lncRNA‐mRNA correlation in diseases, including acute intracerebral haemorrhage,^36^ ischemic cardiomyopathy,^37^ and HCC.^38^ However, the lncRNA‐mRNA correlation network related to ferroptosis in HCC remains unknown. This study's differential gene analysis and Venn diagram reveal three common DEmRNAs (KIF20A, SLC7A11, and PROK2) associated with ferroptosis. The established ferroptosis‐related lncRNA‐mRNA correlation network further identified SLC7A11 and KIF20A as key components in this network. Additionally, we have identified the SLC7A11‐AS1/SLC7A11 pair as exhibiting the strongest correlation.

Subsequently, we verified the predictive accuracy and stability of the SLC7A11‐AS1/SLC7A11 pair, highlighting its importance in assessing patient characteristics such as gender, pathology, Ishak score and tumour size. Notably, patients with high expression of this pair exhibited significantly lower overall survival rates, emphasizing its clinical relevance and prognostic value in HCC. Thus, further exploration of its underlying mechanisms and therapeutic implications is warranted. Furthermore, our study aims to elucidate its specific functions in HCC. Functional enrichment analysis also revealed associations primarily in pathways related to glutamate metabolism (such as GABA‐A receptor activity and GABA receptor activity) and cell membrane transport (such as metal ion transmembrane transporter activity and ligand‐gated channel activity). Therefore, these findings suggest a potential role for the SLC7A11‐AS1/SLC7A11 pair in modulating these processes in HCC, with implications for cancer progression and drug response. Further investigation into these associations is warranted, given SLC7A11's established role in cancer, particularly in oxidative stress and ferroptosis pathways.^30^

Further exploration of the function of SLC7A11‐AS1 in HCC confirmed the synergistic role of SLC7A11‐AS1/SLC7A11 in ferroptosis within HCC, whereby the function of SLC7A11‐AS1 is mediated through targeting SLC7A11. Previous studies have highlighted the regulatory impact of SLC7A11‐AS1 on SLC7A11. Specifically, in gastric cancer, miR‐33a‐5p directly targets the 3′UTR of SLC7A11‐AS1 and SLC7A11, resulting in a notable decrease in the expression of both SLC7A11‐AS1 and SLC7A11.^39^ Studies by Wang et al. have shown that in colorectal cancer, SLC7A11‐AS1 can indirectly upregulate SLC7A11 expression by increasing the nuclear level of nuclear factor erythroid 2‐related factor 2 (NRF2), as NRF2 serves as a transcriptional activator of SLC7A11.^40^ Besides NRF2, activating transcription factor 4 (ATF4)^41^ and activating transcription factor 3 (ATF3)^10^ also act as transcriptional activators of SLC7A11. In contrast, P53 can act as a transcriptional repressor of SLC7A11.^42^ However, further experimental validation is required to ascertain whether SLC7A11‐AS1 influences SLC7A11 expression through these regulatory factors.

Given that SLC7A11‐AS1 overlaps with the 3′UTR of SLC7A11 mRNA in a ‘tail‐to‐tail’ manner, we confirmed the formation of an RNA–RNA duplex between SLC7A11‐AS1 and SLC7A11 mRNA in the overlapping region. As such, this interaction enhances mRNA stability and positively regulates SLC7A11 expression. However, only overexpression of full‐length SLC7A11‐AS1 can increase SLC7A11 expression levels. In contrast, overexpression of the overlapping or non‐overlapping regions of SLC7A11‐AS1 alone cannot achieve this function. Therefore, we speculate that the binding between SLC7A11‐AS1 and SLC7A11 RNA depends on the overlapping region, but the involvement of the non‐overlapping region is still required for its functionality. The reason behind this could be that the duplex formation occurs at the adjacent exons of the poly(A) tail of SLC7A11, and with the help of the RNA–RNA duplex structure and the non‐overlapping region of SLC7A11‐AS1, the poly(A) tail of SLC7A11 is either wrapped or folded in a complex secondary structure, protecting it from ribonucleases or exosome‐mediated degradation.^43^, ^44^ Another hypothesis is that the binding of lncRNA to mRNA competes with microRNA binding sites, enhancing mRNA stability.^45^ The antisense‐mediated cis‐regulatory mechanism, in addition to regulating the stability of sense transcripts, can also involve influencing epigenetic modifications that affect the processing of sense transcripts, including splicing regulation,^46^ polyadenylation regulation,^47^ and the role of m6A methylation.^48^ As an antisense transcript transcribed with SLC7A11, SLC7A11‐AS1 may regulate SLC7A11 through epigenetic modifications, which require further investigation. Furthermore, while previous studies have shown that the lncRNA HEPFAL can regulate the ubiquitination of SLC7A11 protein in HCC,^49^ our study did not find evidence that SLC7A11‐AS1 affects the protein synthesis and degradation of SLC7A11. Therefore, we believe that SLC7A11‐AS1 primarily regulates the expression of SLC7A11 at the RNA level.

We further explored the potential clinical significance of the SLC7A11‐AS1/SLC7A11 pair as a therapeutic target for HCC. Depletion of SLC7A11‐AS1 resulted in reduced SLC7A11 expression, significantly enhancing the inhibitory effect of erastin on tumour growth and promoting ferroptosis. Cancer cells typically exhibit an increased demand for cysteine and GSH to neutralize the elevated ROS levels within the cell. Consequently, this nutritional dependency often necessitates enhanced functionality of SLC7A11.^11^ Furthermore, significant anticancer effects have been observed in various tumours by blocking the SLC7A11‐GSH axis.^15^ In the clinical context, sorafenib, a leading therapy for advanced HCC, eventually induces resistance often associated with suppressed ferroptosis.^7^ Notably, sorafenib and erastin share functions in xCT‐mediated import and ferroptosis promotion.^50^ Hence, it is hypothesized that targeting SLC7A11‐AS1 to reduce SLC7A11 levels and elevate intracellular lipid peroxides might ameliorate sorafenib resistance in cancer therapy. Therefore, targeting the SLC7A11‐AS1/SLC7A11 pair to reduce intracellular GSH levels and promote cancer cell sensitivity to anticancer drugs may represent a promising therapeutic strategy in cancer treatment.

## 5 CONCLUSION

For the first time, our study identified the ferroptosis‐related lncRNA‐mRNA correlation network based on the SLC7A11‐AS1/SLC7A11 pair as a prognostic factor of HCC patients. Moreover, this study elucidates the regulatory mechanisms of SLC7A11‐AS1 and SLC7A11, providing a theoretical foundation of the SLC7A11‐AS1/SLC7A11 pair as a biomarker and therapeutic target for HCC.

## AUTHOR CONTRIBUTIONS

**Xiao Yuan:** Conceptualization (equal); data curation (lead); formal analysis (equal); validation (equal); writing – original draft (lead). **Yida Wang:** Data curation (supporting); formal analysis (equal); software (lead); validation (equal); visualization (lead). **Sitong Jiao:** Formal analysis (equal); investigation (equal); validation (equal). **Huanhuan Gao:** Formal analysis (equal); validation (equal). **Mengqian Zhang:** Formal analysis (supporting); validation (equal). **Xin Wang:** Data curation (equal); validation (equal). **Xunyu Zhou:** Validation (supporting). **Chuanfang Wu:** Conceptualization (equal); funding acquisition (equal); supervision (equal). **Jinku Bao:** Funding acquisition (equal); supervision (equal); writing – review and editing (lead).

## FUNDING INFORMATION

This work was supported by a grant from the National Natural Science Foundation of China [No. 31971162, 32,071,275 and U20A20410].

## CONFLICT OF INTEREST STATEMENT

The authors declare that they have no known competing financial interests or personal relationships that could have appeared to influence the work reported in this paper.

## ETHICS STATEMENT

Ethics Committee approval was obtained from the Institutional Ethics Committee of Sichuan university to the commencement of the study.

## CONSENT

The paper has not been previously published in any other journal or conference proceedings and is not currently under consideration for publication elsewhere.

## Supporting information


Figure S1.
Figure S2.
Figure S3.
Figure S4.
Figure S5.
Figure S6.
Figure S7.

Table S1.
Table S2.
Table S3.

## Data Availability

The datasets used and/or analysed during the current study are available from the corresponding author on reasonable request.
