# Supplementary material for: Identification of SLC7A11‐AS1/SLC7A11 pair as a ferroptosis‐related therapeutic target for hepatocellular carcinoma
Source: J Cell Mol Med. 2024 Jul 10;28(13):e18496. doi: 10.1111/jcmm.18496 (PMC11234646; doi:10.1111/jcmm.18496)
Supplement: Supplementary file 1 — Figure S1. Figure S2. Figure S3. Figure S4. Figure S5. Figure S6. Figure S7. [file JCMM-28-e18496-s001.docx]

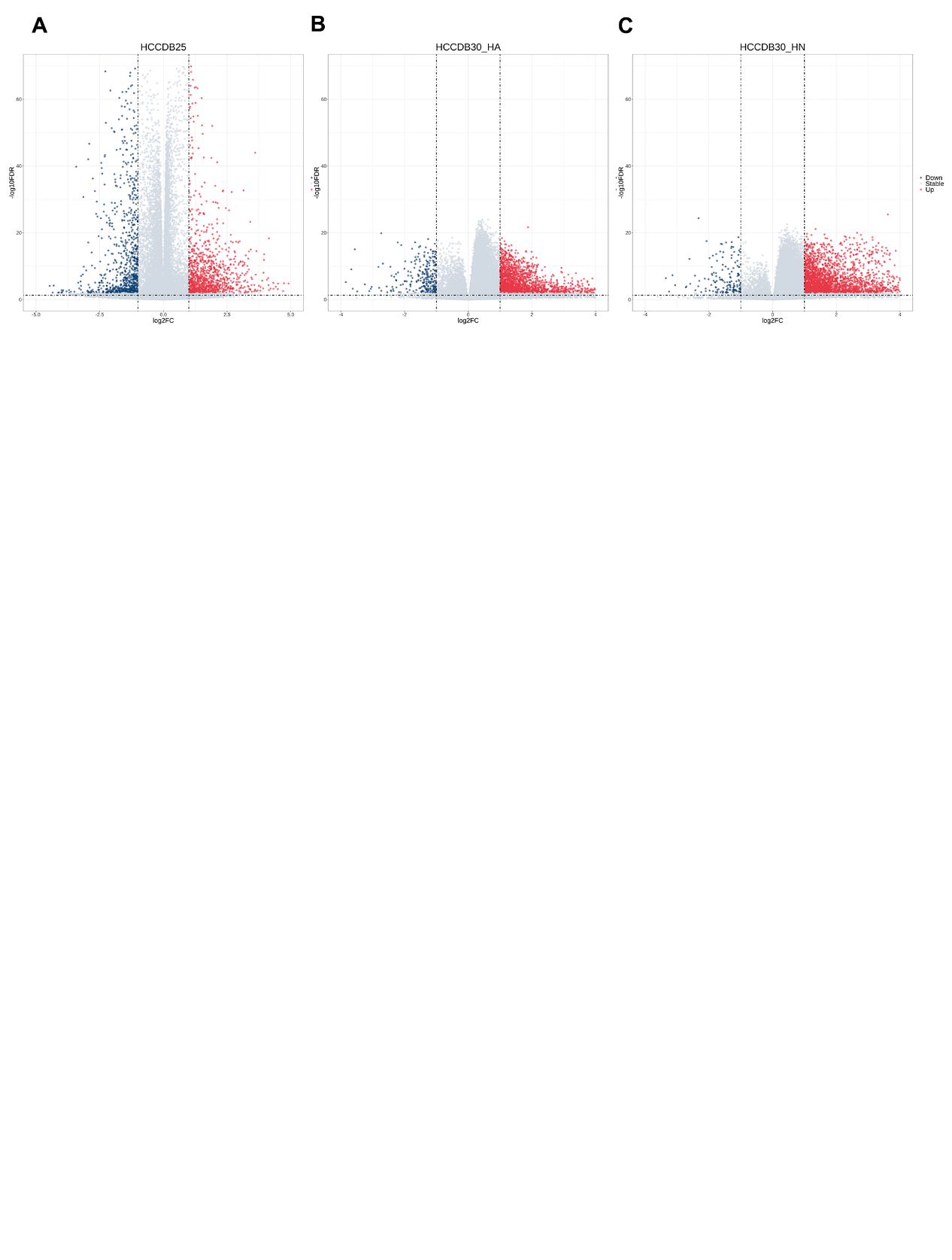


Supplementary figure 1 Identification of differentially expressed genes in HCC patients. (**A-C**) Volcano plots illustrate the differentially expressed genes in HCCDB25 (A), HCCDB30_HA (B), and HCCDB30_HN (C).


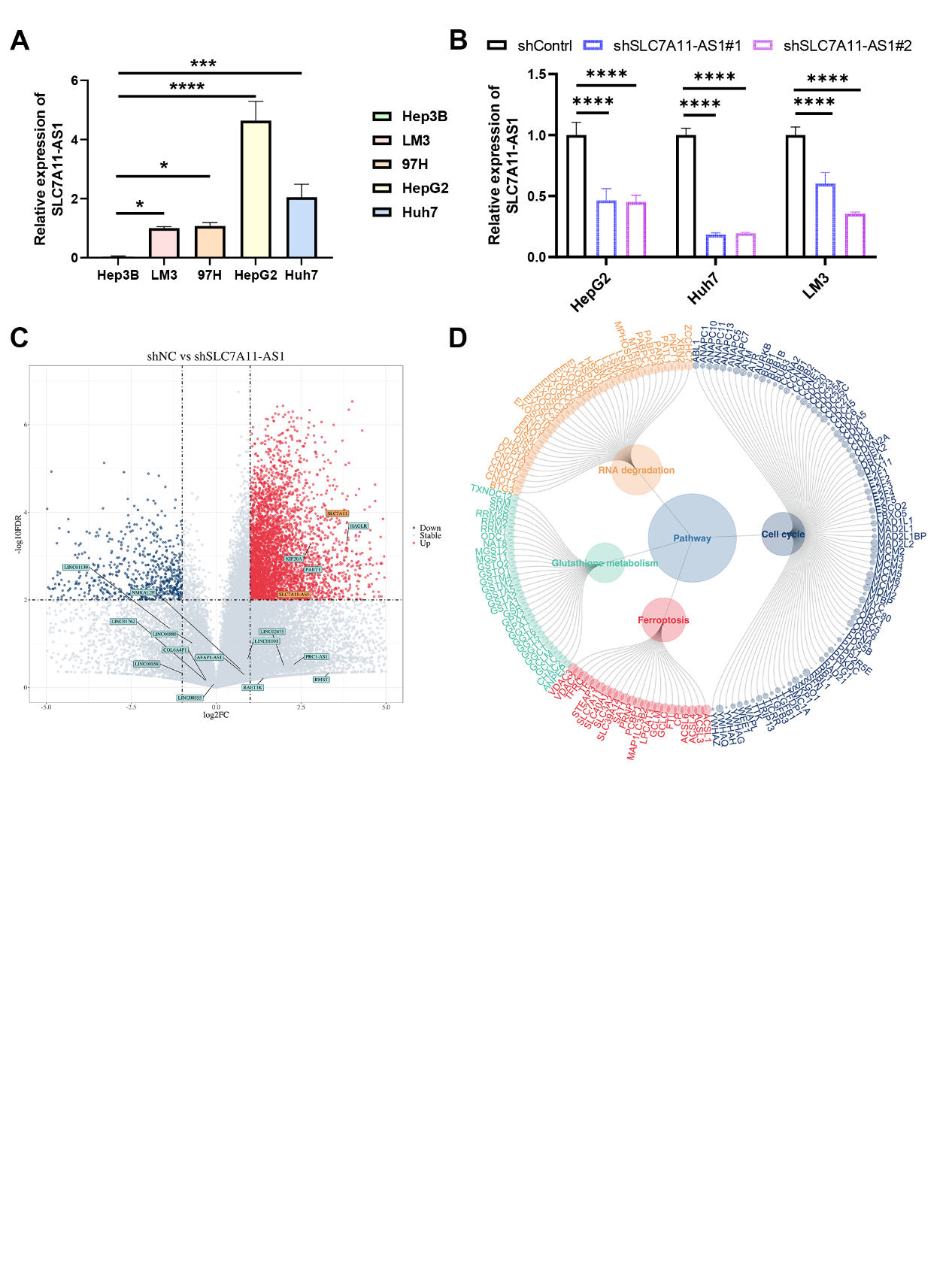


Supplementary figure 2 Differentially Expressed Genes and Functional Enrichment Analysis of RNA-seq. (**A**) The expression of SLC7A11-AS1 in hepatic cell line and 5 different hepatocellular cell lines was detected by qRT-PCR. (**B**) The expression of SLC7A11-AS1 in HepG2, HuH7, and LM3 cells after knockdown of SLC7A11-AS1. (**C**) The volcano plot illustrates the differentially expressed genes following the knockdown of SLC7A11-AS1. (**D**) Petal plots depict the differentially expressed genes involved in RNA degradation, glutathione metabolism, ferroptosis, and the cell cycle.


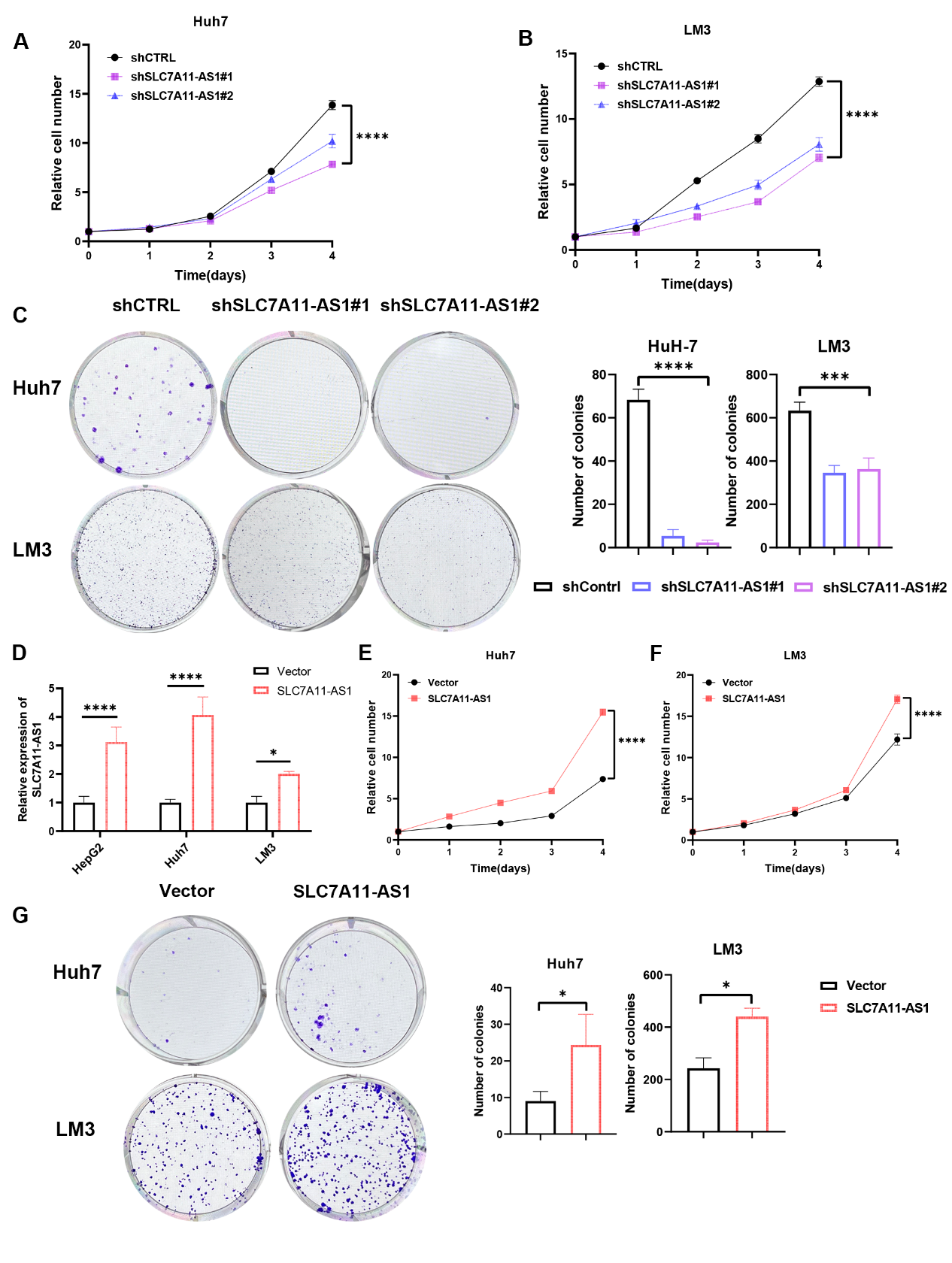


Supplementary figure 3 SLC7A11-AS1 promotes cell proliferation in Huh7 and LM3. (**A-B**) The CCK-8 assay was used to assess cell proliferation rate following the knockdown of SLC7A11-AS1 in Huh7 (A) and LM3 (B). (**C**) The clonogenic assay was employed to assess the clonogenic formation ability in Huh7 and LM3. (**D**) The efficiency of overexpressing SLC7A11-AS1 was assessed using RT-qPCR. (**E-F**) The CCK-8 assay was used to assess cell proliferation rate following the overexpression of SLC7A11-AS1 in Huh7 (E) and LM3 (F). (**G**) The clonogenic assay was employed to assess the clonogenic formation ability in Huh7 and LM3. Data are presented as mean ± S.D. of three independent experiments. *p<0.05,**p<0.01,***p＜0.001，****p＜0.0001，ns: not significant.


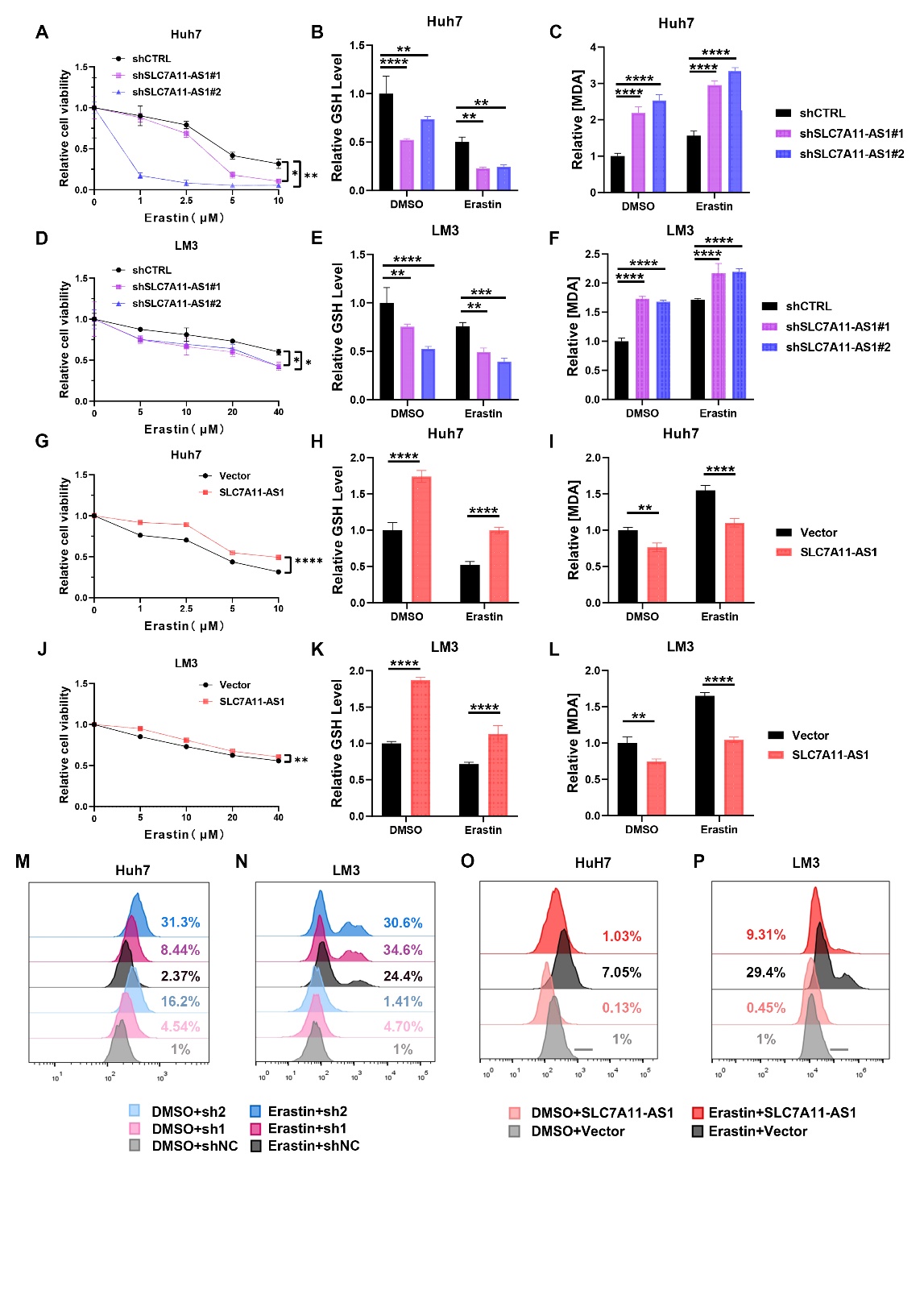


Supplementary figure 4 SLC7A11-AS1 suppresses ferroptosis in Huh7 and LM3. (**A**) The CCK-8 assay was employed to evaluate the toxicity of erastin at various concentrations on Huh7 cells after knocking down of SLC7A11-AS1. (**B-C**) After 24 hours of erastin treatment, the levels of GSH (B) and MDA (C) were measured in Huh7 cells afer knocking down of SLC7A11-AS1. (**D**) The CCK-8 assay was employed to evaluate the toxicity of erastin at various concentrations on LM3 cells after knocking down of SLC7A11-AS1. (**E-F**) After 24 hours of erastin treatment, the levels of GSH (E) and MDA (F) were measured in LM3 cells afer knocking down of SLC7A11-AS1. (**G**) The CCK-8 assay was employed to evaluate the toxicity of erastin at various concentrations on Huh7 cells after overexpression of SLC7A11-AS1. (**H-I**) After 24 hours of erastin treatment, the levels of GSH (H) and MDA (I) were measured in Huh7 cells after overexpression of SLC7A11-AS1. (**J**) The CCK-8 assay was employed to evaluate the toxicity of erastin at various concentrations on LM3 cells after overexpression of SLC7A11-AS1. (**K-L**) After 24 hours of erastin treatment, the levels of GSH (K) and MDA (L) were measured in LM3 cells after overexpression of SLC7A11-AS1. (**M-N**) Flow cytometry was employed to assess the levels of L-ROS in Huh7 (M) and LM3 (N) cells following the knockdown of SLC7A11-AS1. (**O-P**) Flow cytometry was employed to assess the levels of L-ROS in Huh7 (O) and LM3 (P) cells following the overexpression of SLC7A11-AS1. Data are presented as mean ± S.D. of three independent experiments. *p<0.05,**p<0.01,***p＜0.001，****p＜0.0001，ns: not significant.


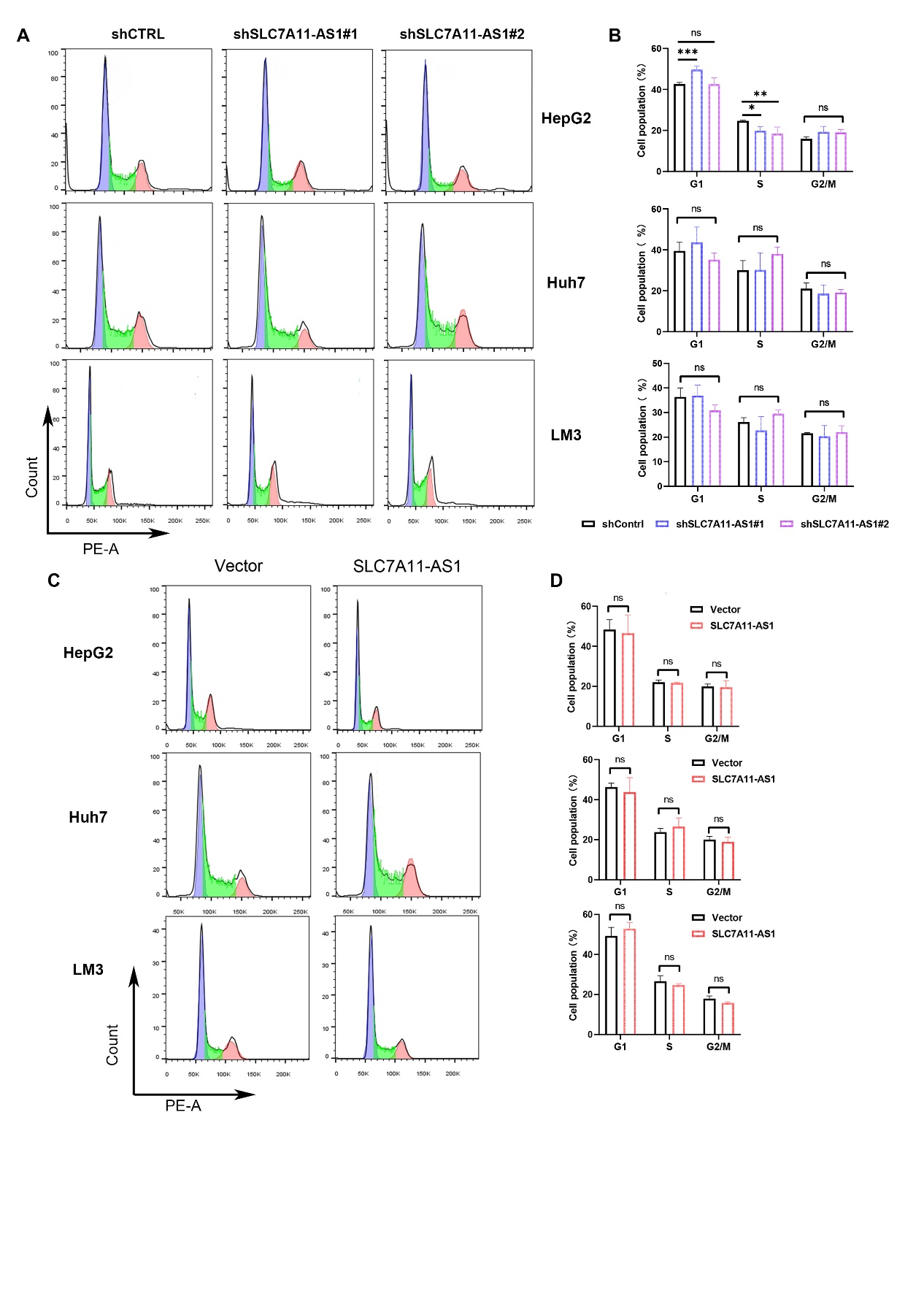


Supplementary figure 5 Absence of Observable Influence of SLC7A11-AS1 on the Cell Cycle in HCC Cells. (**A-B**) Flow cytometry was utilized to assess the cell cycle of HCC cells following the knockdown of SLC7A11-AS1. (**C-D**) Flow cytometry was utilized to assess the cell cycle of HCC cells following the knockdown of SLC7A11-AS1. Data are presented as mean ± S.D. of three independent experiments. *p<0.05,**p<0.01,***p＜0.001，****p＜0.0001，ns: not significant.


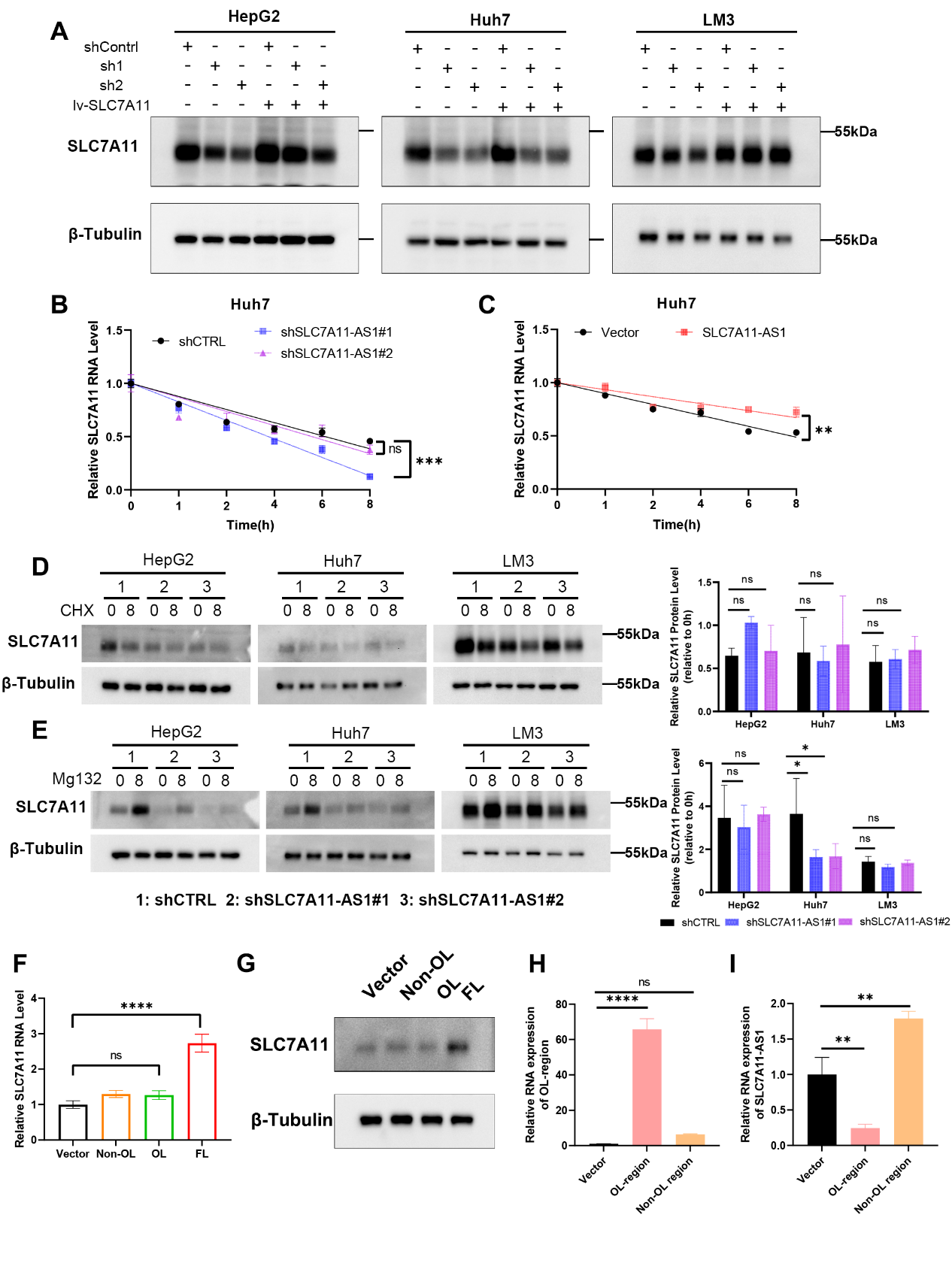


Supplementary figure 7 SLC7A11-AS1 stabilized SLC7A11 mRNA via RNA‐RNA interaction. (**A**) The Western blot assay detected SLC7A11 protein levels in cells with SLC7A11-AS1 knockdown and subsequent SLC7A11 overexpression. (**B-C**) The stability of SLC7A11 mRNA over time was detected by qRT-PCR relative to time 0 after blocking new RNA synthesis with Actinomycin D (20 µg/ml) in dHuH7 cells after knockdown of SLC7A11-AS1(B) or overexpression of SLC7A11-AS1(C). (**D**) After CHX treatment for 0 or 8 hours, Western blot assay was used to measure the protein levels of SLC7A11. (**E**) After MG132 treatment for 0 or 8 hours, Western blot assay was used to measure the protein levels of SLC7A11. (**F-G**) The RNA and protein expression of SLC7A11 in HepG2 cells after overexpression of non-O.L., O.L., or F.L. region of SLC7A11-AS1 by qRT-PCR(F) and western blot analysis(G). (**H-I**) RT-qPCR was used to measure the RNA levels in the O.L. region (H) or non-O.L.region (I) of SLC7A11-AS1. Data are presented as mean ± S.D. of three independent experiments. *p<0.05,**p<0.01,***p＜0.001，****p＜0.0001，ns: not significant.
