# Supplementary material for: Identification of SLC7A11‐AS1/SLC7A11 pair as a ferroptosis‐related therapeutic target for hepatocellular carcinoma
Source: J Cell Mol Med. 2024 Jul 10;28(13):e18496. doi: 10.1111/jcmm.18496 (PMC11234646; doi:10.1111/jcmm.18496)
Supplement: Supplementary file 2 — Table S1. Table S2. Table S3. [file JCMM-28-e18496-s002.docx]

**Supplementary Table 1 Name and sequences of the primers.**

|  | Name | Sequence | |
| --- | --- | --- | --- |
| PCR | OL region-1 | F | ctaccggactcagatctcgagGGCTGAGGAGCTACAGTCTAAATGC |
|  |  | R | gagtcgcgggatcccgggcccCTTATTCTTGTCCTTCTTTAGAAGAGTAAAG |
|  | OL region-2 | F | ctaccggactcagatctcgagAATTGAAAACACTTTTCACAGTATAGACA |
|  |  | R | gagtcgcgggatcccgggcccCTATTGGGGTTAGGAGAAAAGACTAGA |
|  | SLC7A11 | F | CGGAATTCGCCACCatggtcagaaagcctgttgtgt |
|  |  | R | GCTCTAGAtcaCTTATCGTCGTCATCCTTGTAATCtaacttatcttcttctgg |
| shRNA | shContrl | | CCTAAGGTTAAGTCGCCCTCG |
|  | shSLC7A11-AS1#1 | | AAGAGCAGCACTTACTATCAG |
|  | shSLC7A11-AS1#2 | | AAGAAGCTGGCTTACCAACAT |
| RT-qPCR | SLC7A11-AS1 | F | CTCAGCAGCCTTTCACCATT |
|  |  | R | CCTGTCCCTTCTCCCAACTA |
|  | SLC7A11 | F | TCCTGCTTTGGCTCCATGAACG |
|  |  | R | AGAGGAGTGTGCTTGCGGACAT |
|  | β-actin | F | CATGTACGTTGCTATCCAGGC |
|  |  | R | CTCCTTAATGTCACGCACGAT |
|  | 18S | F | ACACGGACAGGATTGACAGA |
|  |  | R | GGACATCTAAGGGCATCACA |
|  | U6 | F | CTCGCTTCGGCAGCACA |
|  |  | R | AACGCTTCACGAATTTGCGT |
|  | OL region-1 | F | GGCTGAGGAGCTACAGTCTAAATGC |
|  |  | R | CTTATTCTTGTCCTTCTTTAGAAGAGTAAAG |
|  | OL region-2 | F | AATTGAAAACACTTTTCACAGTATAGACA |
|  |  | R | CTATTGGGGTTAGGAGAAAAGACTAGA |
| FISH | SLC7A11 FISH probe 1 | | T+TA+TGAGGAGT+TCCACCCAGAC |
|  | SLC7A11 FISH probe 2 | | CAACAAAGA+TCGGAAC+TGCTAA+TG |
|  | SLC7A11 FISH probe 3 | | CCTG+TACTAAA+TGGGTCCGAA+TA |
|  | SLC7A11-AS1 FISH probe 1 | | AGGCAG+TA+TCAGCAGGGTC+TG |
|  | SLC7A11-AS1 FISH probe 2 | | TC+TGTCTGTC+TCCCATAC+TAACCA |
|  | SLC7A11-AS1 FISH probe 3 | | T+TCCCAAG+TCCAGA+TGAGAACAC |
| RPA | OL region-1 | F | CTTTACTCTTCTAAAGAAGGACAAGAATAAG |
|  |  | R | GCATTTAGACTGTAGCTCCTCAGCC |
|  | OL region-2 | F | TCTAGTCTTTTCTCCTAACCCCAATAG |
|  |  | R | TGTCTATACTGTGAAAAGTGTTTTCAATT |
|  | Non-OL region | F | TCCTGCTTTGGCTCCATGAACG |
|  |  | R | AGAGGAGTGTGCTTGCGGACAT |
|  | SLC7A11-AS1 sense | F | TCTGTCTGGCAAGCGCCGCGCGCAT |
|  |  | R | TAATACGACTCACTATAGGGAGAGGAACAAGACTTTA |
|  | SLC7A11-AS1 antisense | F | TAATACGACTCACTATAGGGTCTGTCTGGCAAGCGCCGCGCGCAT |
|  |  | R | GGGAGAGGAACAAGACTTTAT |

**Supplementary Table 2 Antibody information**

| Protein | Cat No | Company |
| --- | --- | --- |
| SLC7A11(WB) | A2413 | ABclonal |
| SLC7A11(IHC) | 26864-1-AP | Proteintech |
| β-tubulin | AC008 | ABclonal |
| β-actin | AC026 | ABclonal |

**Supplementary Table 3 Interactions and their Pearson correlation coefficients (r) shared in the Ferroptosis-related lncRNA-mRNA network**

| Interaction  (lncRNA-mRNA) | HCCDB25  (r) | HCCDB30_HA  (r) | HCCDB30_HN  (r) | AVR  (r) | p |
| --- | --- | --- | --- | --- | --- |
| SLC7A11-AS1_SLC7A11 | 0.380 | 0.914 | 0.914 | 0.736 | *** |
| LINC01224_KIF20A | 0.696 | 0.646 | 0.596 | 0.646 | *** |
| PRC1-AS1_KIF20A | 0.360 | 0.795 | 0.735 | 0.630 | *** |
| NMRAL2P_SLC7A11 | 0.635 | 0.641 | 0.609 | 0.628 | *** |
| LINC01139_KIF20A | 0.432 | 0.642 | 0.632 | 0.569 | *** |
| LINC01108_KIF20A | 0.473 | 0.634 | 0.588 | 0.565 | *** |
| HAGLR_KIF20A | 0.604 | 0.533 | 0.517 | 0.551 | *** |
| LINC02475_KIF20A | 0.567 | 0.550 | 0.503 | 0.540 | *** |
| LINC01091_KIF20A | 0.391 | 0.576 | 0.558 | 0.508 | *** |
| NMRAL2P_KIF20A | 0.622 | 0.444 | 0.409 | 0.492 | *** |
| RAET1K_KIF20A | 0.423 | 0.523 | 0.508 | 0.485 | *** |
| LINC00880_KIF20A | 0.373 | 0.543 | 0.496 | 0.471 | *** |
| LINC01980_KIF20A | 0.471 | 0.482 | 0.451 | 0.468 | *** |
| ST8SIA6-AS1_KIF20A | 0.434 | 0.508 | 0.458 | 0.466 | *** |
| LINC01762_KIF20A | 0.351 | 0.512 | 0.511 | 0.458 | *** |
| AFAP1-AS1_SLC7A11 | 0.359 | 0.515 | 0.493 | 0.456 | *** |
| LINC00355_KIF20A | 0.487 | 0.441 | 0.424 | 0.451 | *** |
| LINC-ROR_KIF20A | 0.422 | 0.510 | 0.417 | 0.450 | *** |
| LINC01446_KIF20A | 0.422 | 0.473 | 0.441 | 0.446 | *** |
| MIR2052HG_SLC7A11 | 0.477 | 0.434 | 0.373 | 0.428 | *** |
| LINC01980_SLC7A11 | 0.567 | 0.381 | 0.325 | 0.424 | ** |
| MIR2052HG_KIF20A | 0.341 | 0.493 | 0.435 | 0.423 | *** |
| BSN-DT_KIF20A | 0.340 | 0.477 | 0.433 | 0.417 | *** |
| LINC01535_KIF20A | 0.469 | 0.387 | 0.386 | 0.414 | *** |
| LINC00858_KIF20A | 0.333 | 0.424 | 0.437 | 0.398 | *** |
| LINC01419_KIF20A | 0.396 | 0.427 | 0.365 | 0.396 | *** |
| COL6A4P1_KIF20A | 0.406 | 0.402 | 0.374 | 0.394 | *** |
| PART1_KIF20A | 0.302 | 0.436 | 0.421 | 0.386 | *** |
| LINC00648_KIF20A | 0.361 | 0.394 | 0.389 | 0.381 | *** |
| HOXA11-AS_KIF20A | 0.377 | 0.438 | 0.325 | 0.380 | ** |
| CASC20_KIF20A | 0.350 | 0.399 | 0.390 | 0.380 | *** |
| LEF1-AS1_SLC7A11 | 0.308 | 0.436 | 0.393 | 0.379 | *** |
| LINC01762_SLC7A11 | 0.397 | 0.384 | 0.350 | 0.377 | ** |
| LINC00645_KIF20A | 0.311 | 0.410 | 0.376 | 0.366 | *** |
| RMST_KIF20A | 0.385 | 0.388 | 0.322 | 0.365 | ** |

*P<0.05; **P<0.01; ***p<0.001.
